# Spatio-Chromatic Adaptation via Higher-Order Canonical Correlation Analysis of Natural Images

**DOI:** 10.1371/journal.pone.0086481

**Published:** 2014-02-12

**Authors:** Michael U. Gutmann, Valero Laparra, Aapo Hyvärinen, Jesús Malo

**Affiliations:** 1 Department of Mathematics and Statistics, University of Helsinki, Helsinki, Finland; 2 Helsinki Institute for Information Technology, University of Helsinki, Helsinki, Finland; 3 Image Processing Laboratory, Universitat de València, València, Spain; 4 Department of Computer Science, University of Helsinki, Helsinki, Finland; 5 Cognitive Mechanisms Laboratories, ATR, Kyoto, Japan; University of Sussex, United Kingdom

## Abstract

Independent component and canonical correlation analysis are two general-purpose statistical methods with wide applicability. In neuroscience, independent component analysis of chromatic natural images explains the spatio-chromatic structure of primary cortical receptive fields in terms of properties of the visual environment. Canonical correlation analysis explains similarly chromatic adaptation to different illuminations. But, as we show in this paper, neither of the two methods generalizes well to explain both spatio-chromatic processing and adaptation at the same time. We propose a statistical method which combines the desirable properties of independent component and canonical correlation analysis: It finds independent components in each data set which, across the two data sets, are related to each other via linear or higher-order correlations. The new method is as widely applicable as canonical correlation analysis, and also to more than two data sets. We call it higher-order canonical correlation analysis. When applied to chromatic natural images, we found that it provides a single (unified) statistical framework which accounts for both spatio-chromatic processing and adaptation. Filters with spatio-chromatic tuning properties as in the primary visual cortex emerged and corresponding-colors psychophysics was reproduced reasonably well. We used the new method to make a theory-driven testable prediction on how the neural response to colored patterns should change when the illumination changes. We predict shifts in the responses which are comparable to the shifts reported for chromatic contrast habituation.

## Introduction

In this paper, we propose a new method to analyze several data sets jointly and use it to relate properties of chromatic natural images to properties of the primary visual cortex: We show that the new method provides a parsimonious statistical explanation of both spatio-chromatic processing and its adaptation to changes in illumination.

Statistical modeling of natural images under fixed, or uncontrolled, illumination reveals that “Gabor-like” features (oriented, local, bandpass features) are basic building blocks of natural images. These features are robustly obtained if statistical methods are used that take higher than second-order statistical information into account, for instance sparse coding [1], independent component analysis (ICA) and its extensions [2], k-means or restricted Boltzmann machines [3], or maximal causes analysis [4], [5]. If the database of natural images contains chromatic images, features are obtained which are in addition color-opponent, that is blue-yellow, red-green, and white-black [6]–[10]. Color opponency is consistently obtained from tristimulus or hyperspectral images, using both second-order or higher-order approaches [11], [12]. When using ICA, the spatio-chromatic tuning of the learned features was found to be similar to cells in the primary visual cortex (V1) [13]. Depending on the exact assumptions made, some methods yield features which fit experimental data better than others [5], [14], [15].

**Figure 1:**
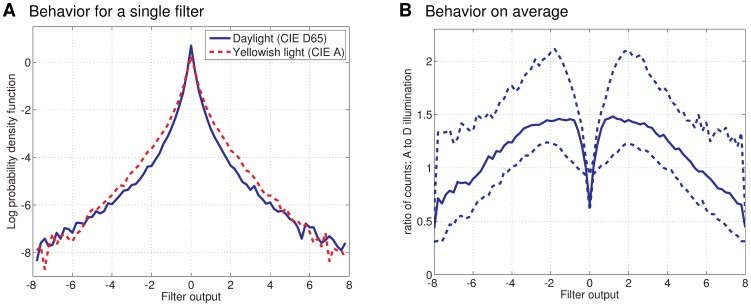
Efficient representations are illumination-dependent. We took ICA filters optimized to illumination CIE D65, daylight, and computed their outputs when the input images are taken under the same illuminant and under illumination CIE A, yellowish light. Each set of images was whitened with optimally adapted whitening filters. We computed histograms for all filter outputs and for both conditions. (a) For a single, randomly chosen filter, we show the log probability density functions (scaled histogram in the log domain) for daylight (blue solid) and yellowish light (red dashed). For yellowish light, the filter output takes more often intermediate values and less often very small ones; the output is less sparse. (b) For each filter, we took the ratio between the histogram obtained for yellowish and daylight illumination. This ratio allows us to read out a loss of efficiency as illumination changes: Since the ratio is smaller than one at zero and for large outputs, the response is less sparse under yellowish light than under daylight. The plot shows the median (solid curve) and the 0.1 and 0.9 quantiles (dashed curves) of the ratios of all filters.

However, the statistical methods in [1]–[14] are not concerned with changing lighting conditions. The same object in daylight radiates a physically different stimulus than indoors under yellowish light. We conducted a simple motivating experiment on how ICA representations are affected by a change in illumination. Figure 1 shows that ICA filters which are optimal for daylight produced less sparse outputs for the same images under yellowish light. This shows that an efficient representation for one illuminant is not necessarily efficient for another one: To maintain efficiency, adaptation of the filters is needed [16].

Statistical modeling of tristimulus pixel values of images under different illuminations provides an explanation of chromatic adaptation for spatially flat stimuli [17]. The cited work explains adaptation in terms of mean and covariance shifts of the tristimulus pixel values. It combines an extension of measurements performed earlier [18] with a decorrelation-oriented explanation of adaptation [19].

However, the statistical methods in [17], [19] are not concerned with the spatial domain, and model second-order chromatic structure (mean and covariance) only. Even after inclusion of spatial information, modeling second-order structure does not yield biologically plausible representations, see Chapter 15 of [2].

Thus, the aforementioned statistical methods account for the different aspects of neural processing in V1 with which they are primary concerned, but neither of these approaches generalizes well to explain both aspects at the same time. We aim here at explaining both spatio-chromatic processing and adaptation using a single statistical method.

**Figure 2:**
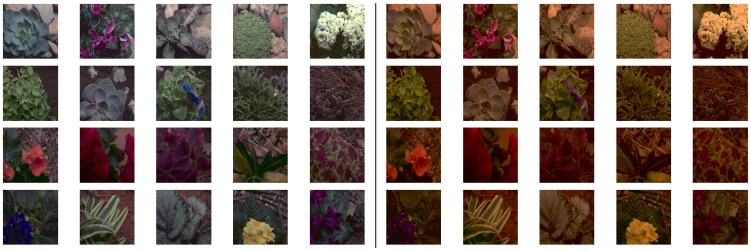
Examples of chromatic images from which we extracted the two data sets used in this paper. The data are image patches 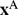 and 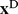 of size 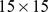 pixels. Left: scenes under CIE D65 illumination from where 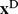 was obtained. Right: the same scenes under CIE A illumination from where 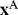 was obtained. Each pair of patches was extracted at the same randomly chosen position.

In this paper, we present a novel statistical method to jointly analyze multiple data sets (a preliminary version was presented before at a conference [20] and applied on video and magnetoencephalography data). The method is a generalization of canonical correlation analysis (CCA) that is sensitive to higher-order statistical structure: It finds independent components in each data set which, across the two data sets, are related to each other via linear or higher-order correlations. The new method is as widely applicable as CCA. We call it higher-order canonical correlation analysis (HOCCA). HOCCA is applied to a recently established database of natural images which were captured under two different lighting conditions, namely illumination CIE A, yellowish light, and illumination CIE D65, daylight [21]. Figure 2 depicts example images from the database. We show that the new statistical method allows to link both spatio-chromatic processing and adaptation in V1 to properties of natural images.

## Results

Matlab code and data to reproduce the results are available at the homepage of the first author.

### Higher-order canonical correlation analysis

First, we introduce HOCCA, our new statistical method to analyze multiple data sets jointly. We present HOCCA in line with the other parts of the paper: We consider the analysis of two data sets of natural images under different illumination. HOCCA is applicable to other kinds of data as well, and also to more than two data sets. More details on HOCCA can be found in Materials and Methods and Text S1.

#### Purpose of HOCCA

Given two data sets, the purpose of HOCCA is to efficiently represent the data as a superposition of meaningful features which are related to each other.

We denote the random vector corresponding to the first data set by 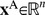, in this paper natural images under illumination CIE A; the random vector corresponding to the second data set is denoted by 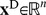, here natural images under illumination CIE D65. We assume that the means have been removed. We also assume that preprocessing consists of individual whitening and, possibly, dimension reduction, both by principal component analysis (see Text S2). We denote the preprocessed data by 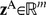 and 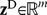, with 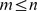.

**Figure 3:**
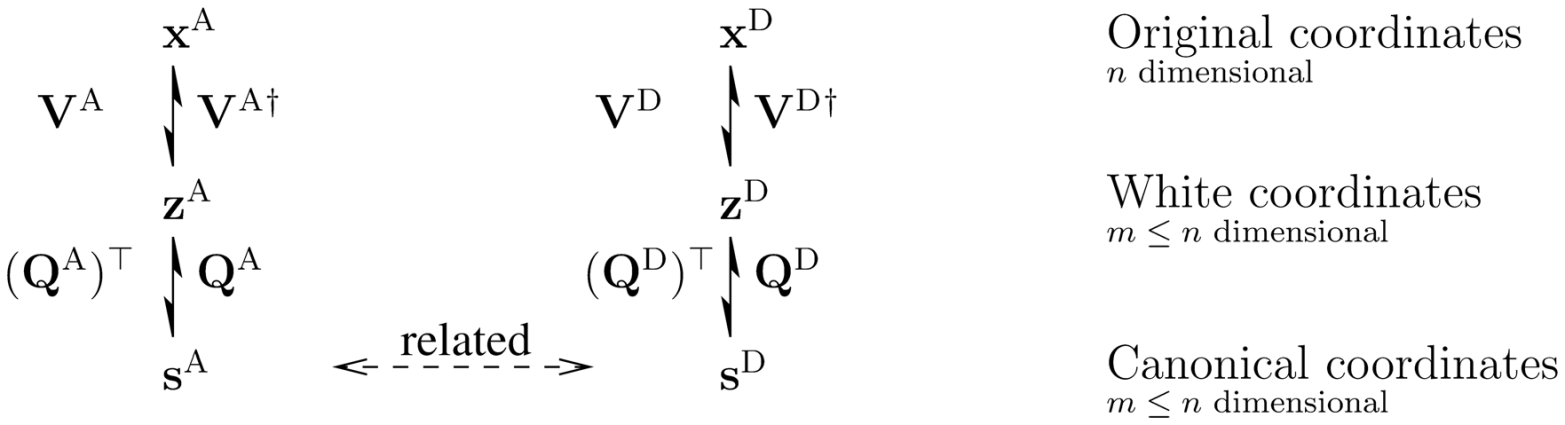
Representing data in terms of coupled canonical coordinates. In this paper, random vectors 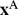 and 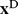 denote natural images under illumination CIE A (yellowish light) and under illumination CIE D65 (daylight), respectively. The whitening matrices 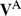 and 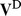 are determined from their covariance matrices. The symbol 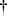 denotes a (pseudo)inverse. See Text S2, Section S2.1, for formulae of these matrices. The purpose of HOCCA is to find the orthogonal matrices 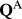 and 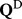 such that, firstly, 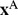 and 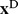 are efficiently represented via the canonical coordinates 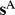 and 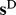, respectively, and that, secondly, the elements 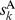 and 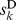 of the vectors 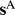 and 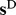 are in a pairwise manner related to each other. We call each row of the compound matrix 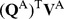 a filter or a sensor, and each column of 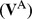 ^†^ 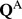, and of 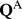 alone, a feature or optimal stimulus. The same naming convention is used for the quantities related to D65 illumination.

With these basic assumptions, the purpose of HOCCA is to represent 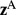 and 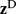 as superpositions of features 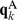 and 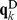,
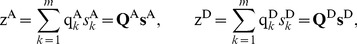
(1)such that, firstly, the canonical coordinates 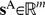 and 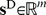 represent the data efficiently and that, secondly, their 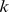-th elements 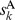 and 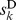 are related to each other. We use the terms “efficient” and “related” here rather loosely. The 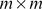 matrices 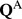 and 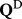 are orthonormal and contain the features as column vectors. Figure 3 summarizes the representation of the data 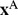 and 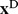 in terms of the canonical coordinates 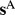 and 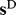, respectively.

Related features exist naturally for the data considered in this paper since the images taken under the different illuminants depict the same physical objects. The statistical dependencies between 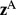 and 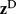 are due the similar reflectance properties of the objects contained in the data sets.

In Text S1 we deal with the more general case where 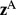 and 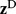 can have different dimensionalities. That is, 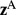 is assumed to have dimension 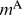 and 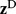 dimension 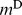. The purpose of HOCCA stays the same. Since we assume that only one 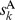 is related to one 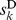, there can only be 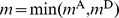 coupled canonical coordinates. The remaining canonical coordinates are “free” and can be used to maximize representation efficiency.

#### Key properties of HOCCA

In order to find a both efficient and related representation of the data, we constructed HOCCA so that higher-order statistical dependencies both within and across the data sets are taken into account. The construction of HOCCA is based on a probabilistic generative model of the data which is explained in Materials and Methods. In brief, the model couples two ICA models, one for 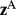 and one for 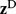, together by assuming that the independent components have statistical dependencies across the two data sets.

HOCCA has the following two key properties:

1. (Efficiency of representation) Sparsity of the estimated canonical coordinates 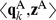 and 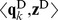 is taken into account when the features 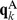 and 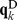 are learned.
2. (Relation between data sets) The canonical coordinates can have linear or higher-order (variance) correlations across the data sets.

In addition to the coupled features 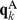 and 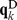, HOCCA yields estimates for the correlation coefficients 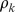 between the canonical coordinates 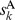 and 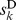. HOCCA also estimates the degree of sparsity 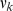 (non-Gaussianity) of the canonical coordinates. Values close to two indicate strong non-Gaussianity while large values indicate an almost Gaussian distribution.

The above properties are in stark contrast to canonical correlation analysis (CCA). CCA represents the data using related features as in (1), but sparsity of the canonical coordinates is not a criterion, and CCA is sensitive to linear correlations between the two data sets only, see Text S2 or Chapter 3 of [22]. CCA has been extended in many ways. While extensions exist which are sensitive to higher-order correlations across the two data sets (for example kernel CCA, see the Discussion section), we are not aware of an extension which combines sensitivity to nonlinear correlations with efficiency of representation.

#### Performing HOCCA

HOCCA is performed by solving an optimization problem. The features 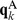 and 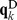, the correlation coefficients 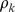 between the canonical coordinates, and the non-Gaussianity indices 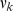 are obtained by maximizing the objective 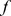,
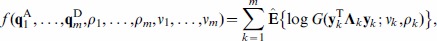
(2)under the constraint that the features of each data set are orthogonal and of unit norm, 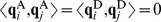 if 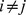 and one if 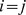. The objective function is based on the log-likelihood of the probabilistic model underlying HOCCA, see Materials and Methods and Text S1. The symbol 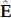 denotes the sample average over the whitened data. The vector 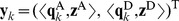 contains the two inner products between the feature vectors and the whitened data 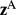 and 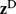. The matrix 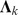 is the precision matrix of the two random variables 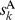 and 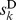 which have unit variance and correlation coefficient 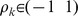,
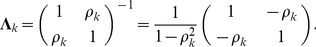
(3)The parametrized function 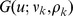 is
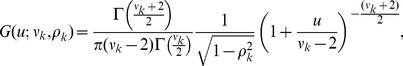
(4)which is valid for 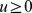 and 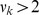.

The objective function 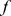 is a sum of 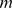 terms where each term only depends on a specific pair of features 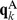 and 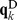. This allows for an optimization scheme where the 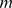 terms are subsequently optimized, under the constraint that the new features 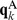 and 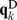 have unit norm and are orthogonal to the previous ones: 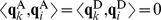, 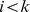. In the simulations in this paper, we used such a sequential optimization.

We show in Text S1 that the objective function 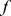 stays valid in the more general setting where the dimensionality of 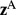 and 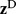 may differ. Maximizing 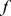 yields the 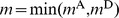 coupled features 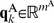 and 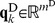, as well as the corresponding 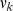 and 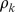.

#### HOCCA as a nonlinear generalization of CCA

We show here that HOCCA is a nonlinear generalization of CCA: For large values of 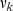, the features which maximize the objective 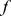 in (2) are those which are obtained with CCA.

The objective in (1) considered as a function of the features is
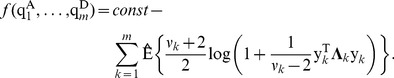
(5)For large 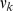 the term 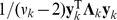 is small so that we can use the first-order Taylor expansion 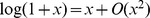. Taking further into account that the data is white and that the features have unit norm, we show in Text S1, Section S1.3, that
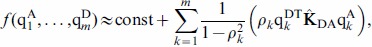
(6)where 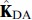 is the sample cross-correlation matrix between 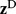 and 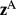. Since 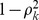 is positive, the objective in (6) is maximized when 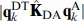 is maximized for all 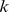 under the orthonormality constraint for the features of each data set. We need the absolute value since 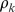 can be positive or negative. This set of optimization problems is the one solved by CCA, up to a possible difference in the signs, see Text S2, Section S2.3. CCA maximizes 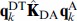 so that for negative 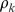, one of the features obtained with the maximization of 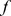 has switched signs compared to the one obtained with CCA.

#### Illustration of HOCCA

We illustrate here properties of HOCCA and provide some intuition by means of a simple example. We assume that 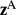 is two dimensional and 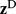 only one dimensional. The example thus demonstrates the applicability of HOCCA to data sets of different dimensionalities. Since the features are orthogonal, 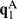 is of the form 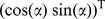, for a certain angle 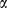, and 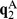 is the vector orthogonal to 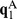. Feature 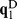 is the scalar one (the sign is arbitrary). In this simple example, 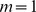 and the sum in (2) collapses to a single term.

**Figure 4:**
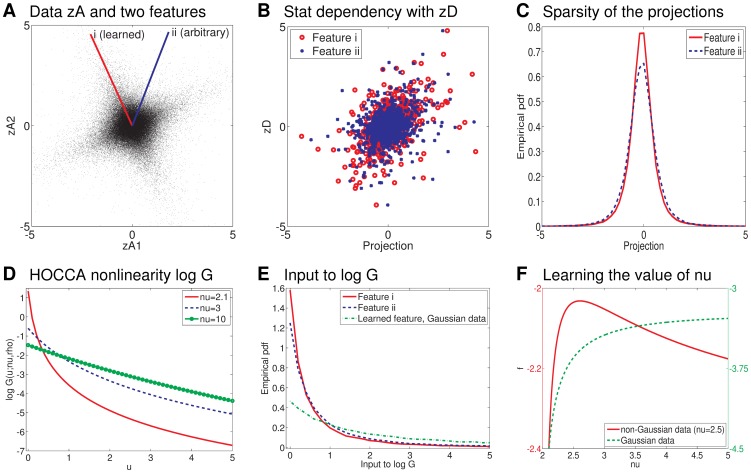
Illustrating HOCCA with a simple example where 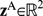 and 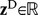. In (a), we show two features 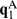 overlaid on the scatter plot. Feature i was learned by HOCCA. Feature ii is an arbitrary alternative feature. Feature i corresponds better to the goals of HOCCA than feature ii since it yields a canonical coordinate (projection) 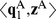 which is more strongly statistically dependent on 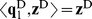 (subfigure b) and also sparser (subfigure c). (d) The nonlinearity 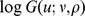 for different values of 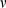 with 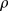 fixed to 0.5. Changing 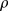 does only lead to an additive offset, it does not change the shape of the nonlinearity. (e) The distribution of the input to 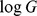, 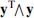, is shown for the two features in (a). We also show the distribution for the feature learned for Gaussian data. In this case, the inputs to the nonlinearity 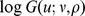 are less often close to zero. (f) The HOCCA objective 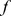 as a function of 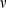 for both non-Gaussian and Gaussian data. For the non-Gaussian data, maximizing 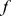 identifies the correct value of 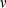. For the Gaussian data, 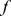 is increasing as 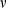 increases (this holds also beyond the range of 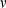 shown here). For large 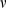 the nonlinearity in (d) is less peaked at zero, which corresponds well to the less peaked distribution for Gaussian data in (e).

We generated data according to the probabilistic model underlying HOCCA (see Materials and Methods and Text 1) with 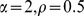, and 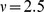. For illustration purposes, the sample size was chosen to be rather large, we used 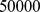 samples. A scatter plot of 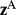 is shown in Figure 4(a). Two features 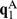 are overlaid on the plot. Feature i was learned by HOCCA. Feature ii is an arbitrary alternative feature. Figure 4(b) shows scatter plots of the canonical coordinates, 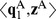 against 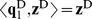, and Figure 4(c) shows the distributions of 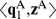 for the features in Figure 3(a). Feature i corresponds better to the goals of HOCCA than feature ii since it yields a canonical coordinate which is sparser and more strongly statistically dependent on 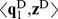. The learned correlation coefficient was 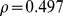. Feature ii gave a correlation coefficient of 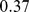.

Computing derivatives shows that 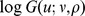 is monotonically decreasing and strictly convex in 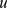. Figure 4(d) shows 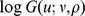 for different values of 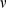 and for 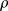 fixed to 0.5. According to the definition of 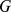 in Equation (4), 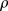 affects 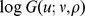 only through the additive offset 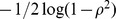 which is increasing as 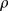 tends to 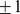. The offset is the mutual information between two Gaussian random variables with correlation coefficient 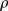 (see Materials and Methods). It provides a mechanism which allows HOCCA to find correlated features.

The argument of 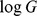 is the quadratic form 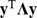 where 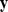 depends on 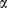 and 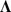 on 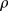. The elements of 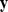 are the estimated canonical coordinates, and 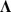 is an estimate of their inverse covariance matrix. The quadratic form 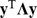 corresponds thus to the squared norm of the estimated canonical coordinates after decorrelation (it is the squared Mahalanobis distance of 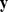 from the origin). Since 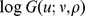 is convex, maximizing 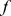 for a fixed value of 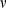 consists in finding features for which the norm of the decorrelated 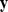 is sparse, see Chapter 6 of [2]. The sum of two squared values is large or close to zero if each of the two decorrelated canonical coordinates are large or close to zero at the same time. This provides a mechanisms which allows HOCCA to find sparse canonical coordinates with possible variance correlations.

Figure 4(e) shows the distribution of 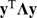 for the two features depicted in Figure 4(a). From a comparison with Figure 4(c), it can be seen that the feature which produces sparser canonical coordinates is also the feature which produces inputs to 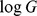 which are more often close to zero, in line with our reasoning above. The figure also shows the distribution of 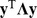 for the learned feature when the data is Gaussian (with 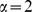 and 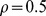 as for the non-Gaussian data above). It can be seen that the inputs to 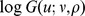 are less often close to zero for that data. The different curves of 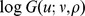 in Figure 4(d) suggest that, for the Gaussian data, the objective 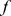 will be larger for 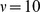 than for 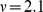.

In HOCCA, the parameter 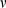 is learned from the data by maximizing 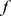. Figure 4(f) shows the HOCCA objective 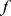 as a function of 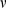, with 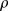 and 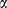 fixed to their true values. We see that for the generated non-Gaussian data, 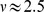 is maximizing 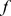 (red solid curve, left axis). The same figure also shows 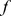 for the Gaussian data (green dashed curve, right axis), where 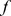 increases as 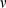 increases. In our numerical optimization, we obtained a value of 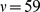, which was the value where our stopping criterion was satisfied. In this regime of 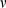, the approximation from the previous section becomes valid, and the features which maximize 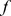 are given by the CCA-features.

#### Validating HOCCA on artificial data

We used artificially generated data to validate HOCCA and to compare it with CCA. We generated data according to (1), with variable levels of correlation and sparsity of the canonical coordinates 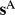 and 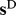, and for randomly generated mixing matrices 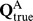 and 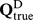 of dimension 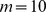. We constructed fifty random estimation problems and used 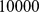 samples to solve them (see Materials and Methods for details). In order to recover the mixing matrices, and thus the features which form their columns, we optimized the objective 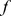 in (2) for HOCCA. For CCA, we solved the singular value problem (S2–7) in Text S2.

**Figure 5:**
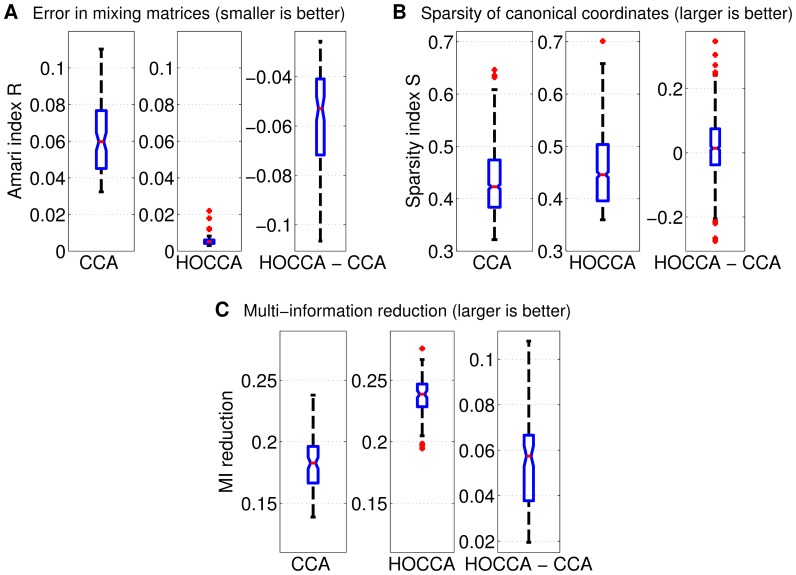
Validating HOCCA on artificial data: Feature identification and representation efficiency. The error of an estimated mixing matrix was measured by the Amari index 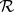 defined in (14). Sparsity of an estimated canonical coordinate was measured using the index 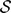 defined in (15). Multi-information reduction was measured by comparing the marginal entropies of the (whitened) data and the estimated canonical coordinates. We show the results for the estimation of 50 random 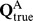 and 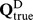 of dimension 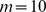: The boxplots in (a) and (c) contain 100 data points each, while the boxplots in (b) show the distribution of all 1000 estimated canonical coordinates. The first and second panel in each subfigure show the distribution of the performance indices for CCA and HOCCA, respectively. The third panel shows the distribution of the difference of the indices. HOCCA recovered the features more accurately, and led to representations with sparser and more independent canonical coordinates than CCA.

We analyzed the results using three measures of performance (see Materials and Methods for details). First, we analyzed how well the mixing matrices (features) are recovered. Figure 5(a) shows that HOCCA led to a better recovery of the mixing matrices. The pointwise comparison in the third panel in the figure shows that HOCCA performed better for each of the fifty random estimation problems.

Second, we analyzed the efficiency of the representation, both from a sparsity and from a related information theoretical point of view. Figure 5(b) shows that the canonical coordinates recovered by HOCCA were mostly sparser than those recovered by CCA – thanks to the active sparsification inherent in HOCCA (the average in the point-wise comparison is larger than zero (one-sided t-test, p-value 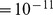)). In line with this result, Figure 5(c) shows that HOCCA led to a stronger multi-information reduction than CCA. The third panel shows that HOCCA led to a more efficient representation for each of the fifty random estimation problems.

**Figure 6:**
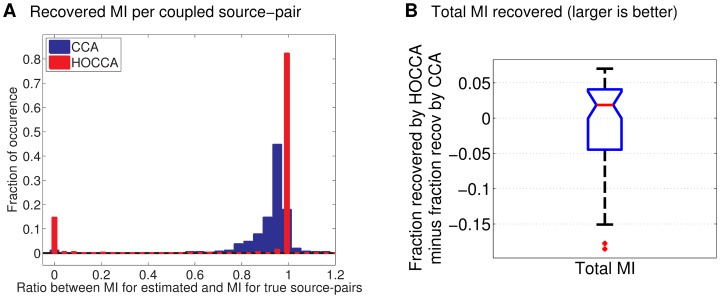
Validating HOCCA on artificial data: Identification of the coupling. (a) We computed the mutual information (MI) between the source-pairs for both the true and the estimated sources, and took their ratio. The distribution of the ratio is bimodal for HOCCA: While the recovery was very accurate in most cases, in some rare cases, the recovered sources were not dependent (local optima). For CCA, the distribution is unimodal: A large amount of the MI was recovered, but the recovered amount was usually smaller than for HOCCA. (b) The boxplot shows the difference between the fraction of total MI that HOCCA can recover per estimation problem and the fraction which CCA can recover. On average, HOCCA recovered more MI between the corresponding sources than CCA. Results for 50 random estimation problems are shown.

Third, we analyzed how well the coupling (correspondence) between the two data sets was identified. For that purpose, we measured the mutual information between the coupled pairs of sources. Figure 6(a) shows that HOCCA recovered in most cases almost all of the mutual information. In some rare cases, however, it failed. In preliminary work, we found that the objective has local optima [20]. The observed failures are presumably due to the fact that the optimization scheme did not find the global maximum. For CCA, such failures were more rare. The mode of the CCA distribution, on the other hand, is smaller than for HOCCA indicating that the general level of recovery was also smaller. In some cases, CCA recovered more mutual information per source-pair than what was actually available. Because the total amount of mutual information between all source-pairs is preserved, this means that CCA over-allocated mutual information for some sources while, consequently, having to allocate less to other sources.

In Figure 6(b), we investigate how much mutual information per estimation problem was recovered. While Figure 6(a) dealt with a comparison per source-pair, this figure is a comparison which takes all the source-pairs per estimation problem into account. The boxplot in the figure shows the difference between the fraction of total mutual information which HOCCA recovered per estimation problem and the fraction which CCA recovered. The distribution is skewed towards positive values which indicates that HOCCA recovered more often more mutual information between the corresponding sources than CCA.

The results reported above validate the theoretical properties of HOCCA: We found that HOCCA led to a more efficient representation of the two data sets than CCA, as measured by sparsity or gain in independence, and that the recovery of the correspondence between the two data sets was also better, as measured by mutual information.

### From natural images to spatio-chromatic adaptation

Next, we apply HOCCA to chromatic natural images that were acquired under two different illumination conditions, daylight and yellowish light. We analyze the learned coupled representations, show that they account for known experimental results and make a theory-driven prediction. Two properties of the learned representations are of particular interest: First, the representation of the two data sets individually, that is, the spatio-chromatic processing for a given illuminant. Second, the coupling (correspondence) between the representations across the two data sets, that is, the adaptation to changes in the illumination. We also compare the representations learned by HOCCA with those from other statistical methods, namely ICA, CCA, and whitening by principal component analysis, see Materials and Methods for details and Tables 1 and 2 for an overview.

**Table 1:** Overview of the methods used to determine the matrices 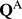 and 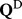 in Figure 3.

Method	Statistics used to determine 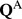 and 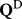
HOCCA	(1) Sparsity of 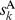 and 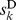
	(2) Correlation and variance dependencies between 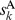 and 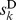
CCA	Correlation between 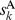 and 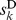
ICA	Sparsity of 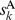 and 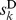 (correspondence determined by postprocessing)
Whitening by PCA	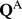 and 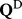 are both the identity matrix (correspondence determined by postprocessing)

The variables 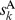 and 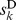 denote the canonical coordinates (feature outputs) of the representations. Higher-order canonical correlation analysis (HOCCA) generalizes canonical correlation analysis (CCA) in terms of the detected dependencies between the canonical coordinates. Moreover, it makes the canonical coordinates sparse which results in an efficient representation of the data. Independent component analysis (ICA) is maximizing the representation efficiency of the individual data sets without taking possible correspondences into account. Whitening by principal component analysis (PCA) is the first processing step in all methods. CCA and HOCCA yield coupled representations. For ICA and whitening, the correspondence between the filter outputs must be determined as part of a postprocessing step. We used mutual information maximization for the matching, see Materials and Methods for details.

**Table 2:** Overview of our comparison of the learned coupled representations of natural images.

Criterion of comparison	Target property	Results
Independence and sparsity of the canonical coordinates	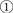	Figure 7
Mutual information between corresponding coordinates	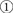	Figure 8
Biological plausibility of the features	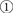	Figures 10, 11
Similarity of the coupled features	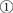	Figures 10, 11, Table 3
Psychophysics of corresponding colors	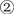	Figures 12, 13, Table 3
Noise-distortion curves	 + 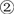	Figure 14

The representations learned by HOCCA, CCA, ICA, and whitening were compared from both statistical and biological points of view using multiple criteria. Two properties of the learned representations are of particular interest 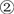 The individual representations of the two data sets, which is related to spatio-chromatic processing for a given illumination condition. 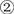 The coupling (correspondence) between the two representations, which is related to adaptation to changes in the illumination. The different criteria measure different aspects of these two properties.

#### Statistical approach to spatio-chromatic adaptation

Applying HOCCA, or one of the alternative methods considered, to the two sets of images produces two sets of coupled filters (sensors), each one adapted to one of the two lighting conditions. The filter outputs yield an internal representation of the images in terms of canonical coordinates, see Figure 3. There is a one-to-one correspondence between the canonical coordinates of each condition, and the corresponding coordinates are statistically dependent. The same one-to-one correspondence applies to the filters and features. The learned correspondence provides a model for spatio-chromatic adaptation: As the illumination changes, the filters should optimally change into their counterparts. The two corresponding filters may be considered to be instances of the same (hypothetical) physical sensor when adapted to the two different illuminants. The internal representation of an image can be adapted to changing lighting conditions by moving from one set of canonical coordinates to the other one.

#### Statistical properties of the learned representations

We analyzed the learned representations of natural images statistically using the same measure as for the artificial data. We used multi-information reduction and sparsity to assess the individual representation of each data set; to assess the coupling we used mutual information between the coupled canonical coordinates.

**Figure 7:**
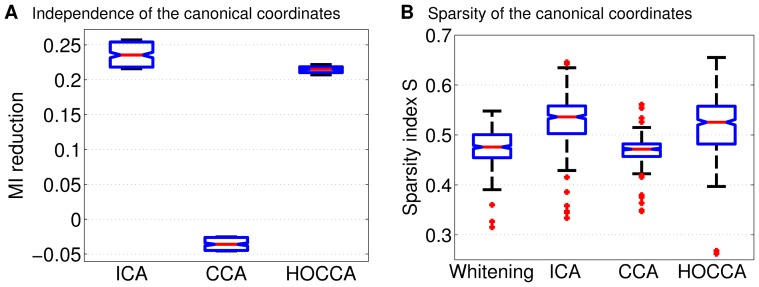
Analyzing the efficiency of the learned representations of natural images using independence and sparsity. (a) Independence was measured using reduction in multi-information (in bits per dimension, relative to whitened and dimensionality reduced data). The boxplot shows the distribution of the reduction for 100 pairs of bootstrapped data sets of size 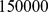. For reference, the multi-information reduction per dimension obtained by whitening without dimensionality reduction was 6.05 bits/dimension. Since dimensionality reduction introduces some information loss, the total reduction with regard to the pixel domain is not the sum of 6.05 bits/dimension plus the reductions reported in the figure. (b) Sparsity was measured using 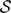 in (15). A Gaussian has a value of 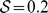. The boxplot shows the distribution of the sparsity of the 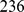 filters learned from natural images under illumination CIE A and CIE D65. The reported sparsity is the average value obtained for the above 100 bootstrapped data sets. The results for the CIE A and CIE D65 data are shown in the same boxplot. The figure suggest that HOCCA resulted in a similarly efficient representation as ICA, and in a more efficient one than CCA, or whitening.

Figure 7(a) shows the amount by which multi-information was reduced by ICA, CCA, and HOCCA after whitening and dimensionality reduction. This means that we compared the reduction achieved by the different methods relative to the reduction achieved by whitening. The figure shows that ICA and HOCCA yielded similar results in multi-information reduction, with ICA being slightly better than HOCCA. Both methods led to a larger reduction than CCA. For CCA, we obtained negative values of multi-information reduction which means that it actually increased the statistical dependencies (redundancy) among the canonical coordinates.

Figure 7(b) shows the sparsity of the canonical coordinates. HOCCA led to a sparser representation than CCA or whitening, and to a slightly less sparse representation than ICA. With another measure of sparsity, robust kurtosis 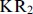 due to J.J.A. Moors [23], we obtained similar results but HOCCA had higher values than ICA (results not shown). The two measures of efficiency shown in Figure 7 are consistent with each other: HOCCA resulted in a similarly efficient representation as ICA, and in a more efficient one than CCA, or whitening.

**Figure 8:**
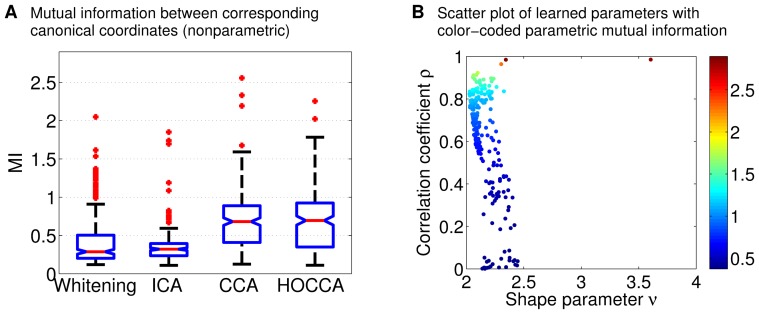
Analyzing the coupling of the learned representations of natural images using mutual information. (a) The nonparametric mutual information (MI) measurement was performed as for the artificial data, using CIE A and CIE D65 data sets of size 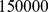. (b) The parametric measurement was performed using (13), see Materials and Methods. The correspondence learned by CCA and HOCCA resulted in coupled canonical coordinates which are more related to each other than the coupled coordinates obtained via ICA or whitening.

Figure 8(a) shows the mutual information between the coupled canonical coordinates. The correspondence learned by CCA and HOCCA resulted in coupled canonical coordinates which are more related to each other than the coupled coordinates obtained via ICA or whitening, as measured by mutual information. This suggests that learning the coupling and the features jointly leads to a stronger coupling than first learning the features and then selecting corresponding pairs by greedily maximizing mutual information.

**Figure 9:**
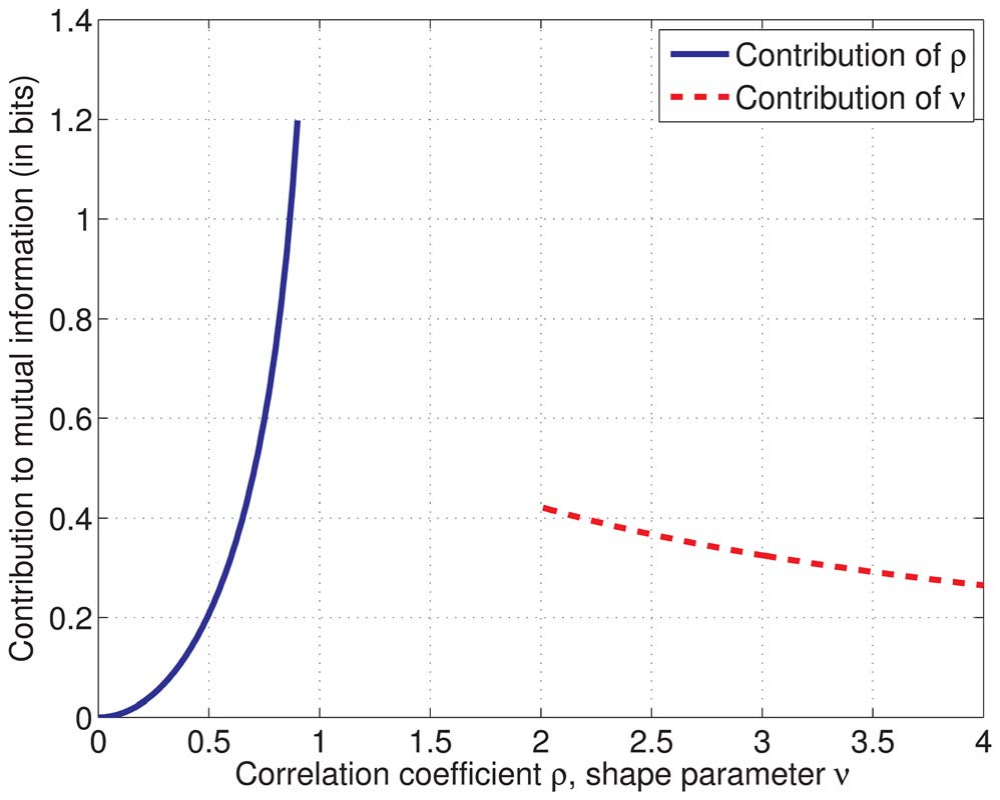
Mutual information for a bivariate student's t-distribution. The correlation coefficient 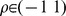 and the shape parameter 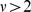 contribute separately to the mutual information, see (13). The contribution of 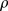 is symmetric around zero and shown in blue for 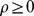 (solid curve), the contribution of 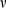 is shown in red (dashed curve). The mutual information of the bivariate student's t-distribution is given by the sum of the two contributions. The contribution of 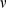 reflects the higher-order statistical dependencies between the two random variables.

Figure 8(b) shows a scatter plot of the learned correlation coefficient 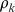 and shape parameter 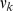 of HOCCA. According to the probabilistic model underlying HOCCA, the mutual information between the coupled canonical coordinates 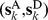 is a function of these parameters, see (13) in Materials and Methods and Figure 9. As the referenced equation and figure show, the two parameters contribute to the mutual information separately. The color of the markers in the figure indicates the value which the mutual information takes for each 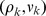. This measurement of mutual information is based on the statistical model underlying HOCCA while in Figure 8(a), mutual information is measured in a nonparametric way. We found that the parametric and nonparametric measurements are consistent with each other (detailed analysis not shown). More importantly, most shape parameters 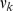 are between 2 and 2.5. With Figure 9, the shape parameters contribute around 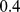 bits to the mutual information, which corresponds to a correlation coefficient of about 0.65 for Gaussian variables. The values of 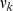 imply, first, that canonical coordinates for which 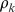 is close to zero are not statistically independent, and second, that their marginal distribution has heavier tails than a Gaussian. This is in line with the sparsity results shown in Figure 7(b).

Taken together, Figures 7 and 8 illustrate that HOCCA combines the desirable efficiency property of ICA with the desirable correspondence property of CCA.

#### The features of the learned representations

**Figure 10:**
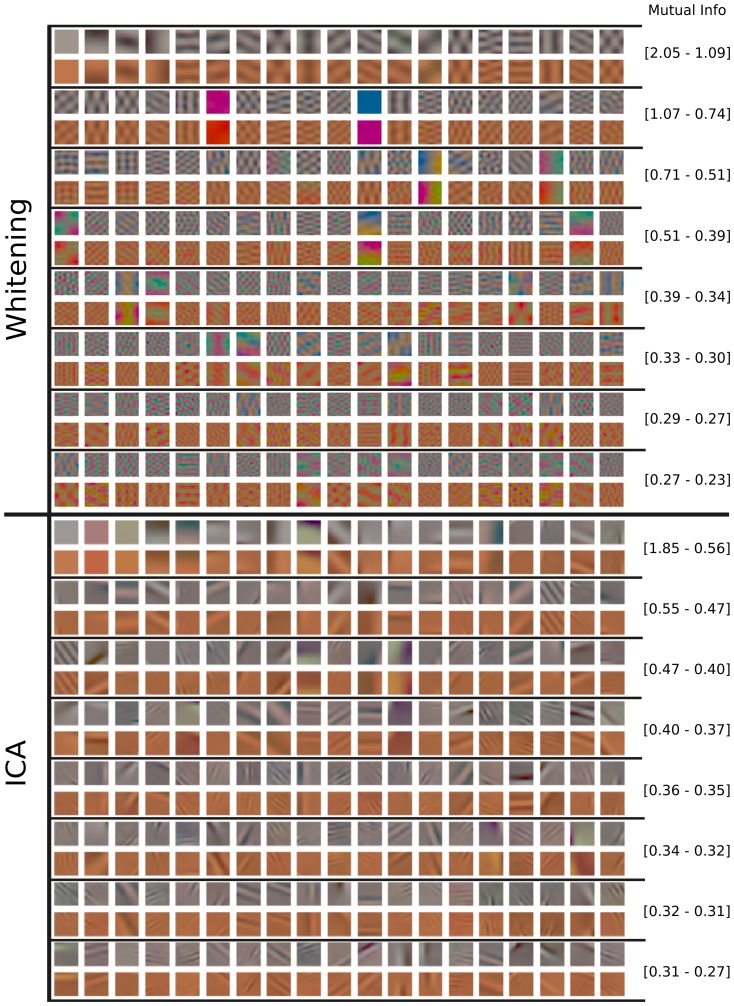
Features learned by whitening and ICA from natural images. After learning, the features from the two data sets were matched so that the mutual information between the corresponding canonical coordinates is maximized, see Materials and Methods for details. In each row, the upper feature is for CIE D65 illumination while the lower feature is the corresponding one for illumination CIE A. Only the first 152 feature-pairs are shown. The feature-pairs are sorted by mutual information between the canonical coordinates. The numbers on the right indicate the range of the mutual information (in bits) for the feature-pairs in each row. ICA features are biologically plausible while whitening features are not.

**Figure 11 pone-0086481-g011:**
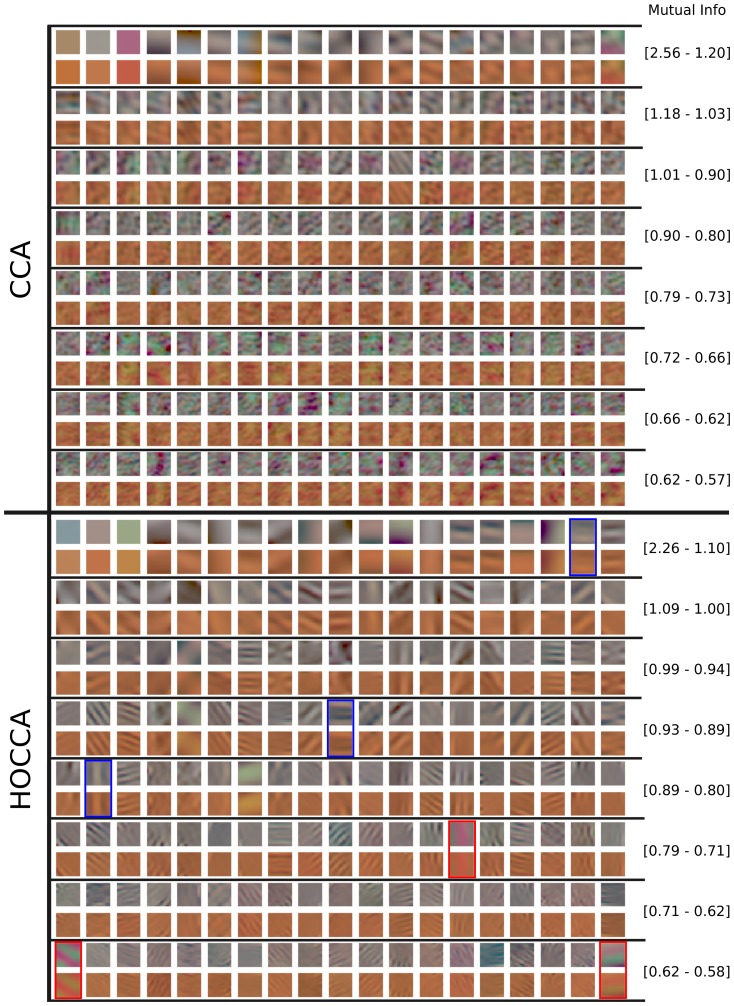
Features learned by CCA and HOCCA from natural images. Only the first 152 feature-pairs are shown. The numbers on the right indicate the range of the mutual information (in bits) for the features in each row. The feature-pairs are arranged as in Figure 10. HOCCA features are biologically plausible while CCA features are not. The pairs of HOCCA features marked with red and blue frames are used to make a prediction about response-adaptation for spatio-chromatic inputs.

Figures 10 and 11 show the first 152 pairs of features which were learned with the different methods. For each pair, the upper feature is for CIE D65 illumination while the lower feature is for illumination CIE A. The feature-pairs are sorted by mutual information between the corresponding canonical coordinates: More related feature-pairs come first. The values of mutual information displayed in the figures indicate the range for the filters in each row. We first analyze the features per data set. Then, we analyze the coupling between the features.

Regarding the features per data set, we use the finding that neurons in V1 are dominantly tuned to spatially localized oriented Gabor-like features with achromatic, red-green and yellow-blue chromatic content as plausibility baseline [24]–[26]. For all methods, the features learned from images under illumination CIE A have oscillations around a yellowish mean, which is reasonable given the yellowish illumination. For the images under illumination CIE D65, the learned features have achromatic averages. Whitening yielded spatially extended gratings of different orientation, frequency and opponent chromatic content, similar to a discrete cosine transform. CCA yielded some low-frequency features, the remaining features show non-localized high frequency oscillations, and have a quite undefined spatial structure. ICA and HOCCA yielded localized Gabor-like features of different orientation, frequency and opponent chromatic content.

Visual inspection of the features shows that for whitening and CCA, the achromatic and the chromatic oscillations in the high frequency features do often not match spatially. They have different fundamental frequencies. For ICA and HOCCA, however, there is no such mismatch between achromatic and chromatic parts. Further, the ICA and HOCCA filters seem chromatically less saturated than the whitening and CCA filters. This means that in order to elicit a comparable response, ICA and HOCCA filters would require a stronger amplitude for chromatic than for achromatic gratings.

Regarding the coupling, the sorting according to mutual information shows that for ICA, HOCCA and CCA, low-frequency features are more related than high-frequency ones. Further, for non-zero frequencies, the achromatic features are more related than the chromatic ones.

We analyzed the similarity of the corresponding features, using the mean squared error as distance measure. Direct application of this distance would, however, be strongly biased by the chromatic shift due to the different illuminations. Therefore, Von-Kries color compensation [27] was applied to the features of illumination CIE A before computing the mean squared error. The resulting spatio-chromatic distances for the different learning methods are shown in Table 3 (first row). HOCCA yielded feature-pairs which are more similar to each other than the other methods. The same result was also obtained using other color compensations than Von-Kries before computation of the distance, such as CIELab [27].

**Table 3:** Quantification of the results in Figures 10 to [Fig pone-0086481-g011] [Fig pone-0086481-g012] 13.

	Whitening	CCA	ICA	HOCCA
Similarity of coupled features (RMSE)	18.7±4.2	17.5±3.6	17.7±4.6	15.7±4.4
Corresponding colors (relative RMSE)	0.29±0.91	0.046±0.13	0.18±0.60	–

**Figure 12 pone-0086481-g012:**
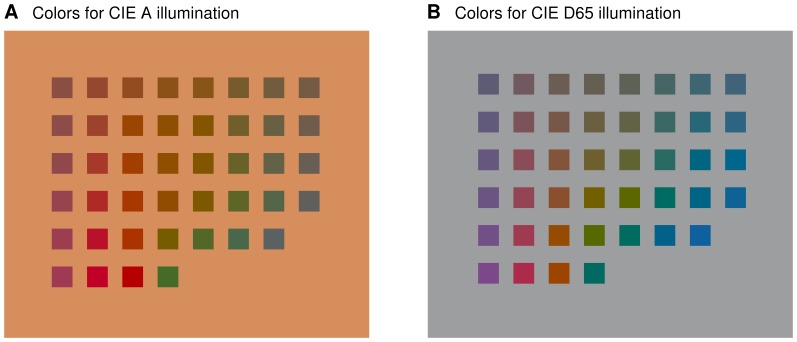
Corresponding-colors psychophysics. For humans, the color of a patch in (a), when seen under CIE A illumination, appears to be the same as the color of the patch in (b) at the same location on the grid, when seen under CIE D65 illumination. Two colors which give rise to the same perception under two different viewing conditions are said to be corresponding. The experimental findings visualized in the figure are due to [30].

**Figure 13:**
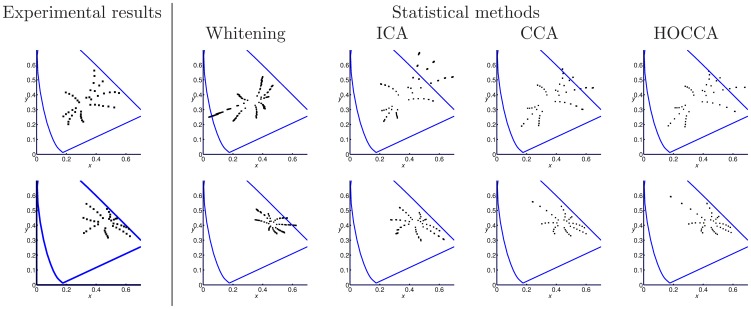
Using the learned representations to reproduce corresponding-colors psychophysics. Left: Experimentally corresponding colors of Figure 12 in the CIE xy diagram. Right, top row: Predictions of the corresponding colors under illumination D65 from samples under illumination A. Right, bottom row: Predictions of the corresponding colors under illumination A from samples under illumination D65.

The numbers indicate the (relative) root mean squared error (RMSE, average 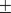 std). First row: Spatio-chromatic similarity between the coupled features in Figures 10 and 11 after Von-Kries color compensation. On average, HOCCA yielded a smaller RMSE than the other methods (two-sample t-test, largest p-value 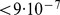). Second row: Prediction error for the color-corresponding pairs in Figures 12 and 13. The RMSE of the different methods is computed relative to the error of HOCCA. A positive relative difference indicates that the alternative method has a larger error than HOCCA. On average, the relative difference is positive for all alternative methods, and significantly larger than zero (one-sided t-test, largest p-value was 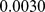).

#### Reproducing psychophysics of corresponding colors

We further investigated the learned coupling by assessing the ability of the different representations to reproduce psychophysical data on color corresponding pairs (color constancy). In the color psychophysics literature, physically different stimuli are referred to as corresponding if they give rise to the same perceived color when viewed under different conditions [28]–[30]. Corresponding colors illustrate the (purely) chromatic adaptation ability of the human visual system and form a standard benchmark for chromatic adaptation models, see, for example, [21].

We show in Figure 12 the experimentally corresponding colors [30]. Figure 13, left column, shows the same corresponding colors in the CIE xy chromaticity diagram. Each point in the lower and upper diagram denotes one color in Figure 12(a) for illumination A and in Figure 12(b) for illumination D65, respectively. In this (standard) visualization, the correspondence between the colors is not made explicit. Qualitative comparisons of different chromatic adaptation models are based on the arrangement of the points in the diagram [21].

Figure 13, right column, shows (linear) predictions of the color-corresponding pairs from the learned representations of the data, performed as described in Materials and Methods. The top row shows the predictions for illumination D65 obtained from the sample colors under illumination A, the bottom row shows the predictions for illumination A obtained from the samples under illumination D65.

A qualitative comparison of these predictions with the experimental data in the left column shows that HOCCA and CCA led to a better performance than whitening or ICA-based correspondence methods: For whitening, the arrangement of the points is rather different from the experimental data. For ICA, the predictions are often over-saturated such that many of the predicted colors fall outside the chromaticity diagram, which means that they are physically unrealistic. For HOCCA and CCA, however, this was much more rarely the case.

A quantitative comparison was performed by computing the root mean squared error between the predicted and the experimental colors in the XYZ color-space. The results are given in Table 3 (second row). Averaging over all color-points, we found that HOCCA gave the best results, followed by CCA. In more detail, we assessed for each color how well the different methods are doing relative to HOCCA. A positive relative difference indicates that the alternative method has a larger root mean squared error. On average, the relative difference was found to be significantly larger than zero for all alternative methods.

#### Adaptation with neural noise constraints

In the previous sections, we used different criteria to analyze the learned representations per data set and the coupling across the data sets. The criteria used assessed the two aspects of the learned representations separately. Here, we consider both aspects at the same time.

The learned filters map the images 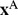 and 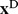 into a canonical domain where they are represented by the coordinates 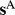 and 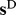. This transformation was considered to be free of noise. Real systems, however, are intrinsically noisy and the noise-level may depend on the signal. A measure of noisiness is the Fano factor 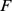 which is the variance of the noise divided by its mean, see Chapter 1 of [31]. In alert Macaque monkeys, the Fano factor in V1 was found to be less than one for optimal stimuli (with an average value of 0.33), and around one for stimuli close to the visual threshold [32].

We investigated how the different representations perform in an adaptation task in the presence of neural noise: We compared the different methods in their ability to predict the representation of an image under illumination CIE D65 from its representation under CIE A. The mean squared prediction error is derived in Materials and Methods and Text S3. It equals
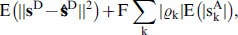
(7)where 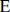 denotes expectation, and where 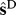 is the prediction when there is no neural noise. The first term is the squared noise-free prediction error. The second term is a weighted sum of sparsity penalties 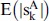. The weighting depends on the Fano factor (noise-level) 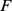 and the correlation coefficients 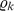 between the canonical coordinates 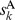 and 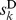. For HOCCA, 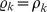. If 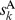 is sparse, the sparsity penalty is small. For 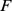 close to zero, the noise-free prediction error dominates but as 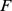 increases, sparsity becomes relevant. For a small overall prediction error, the representations should be sparse and have a good correspondence (coupling).

**Figure 14:**
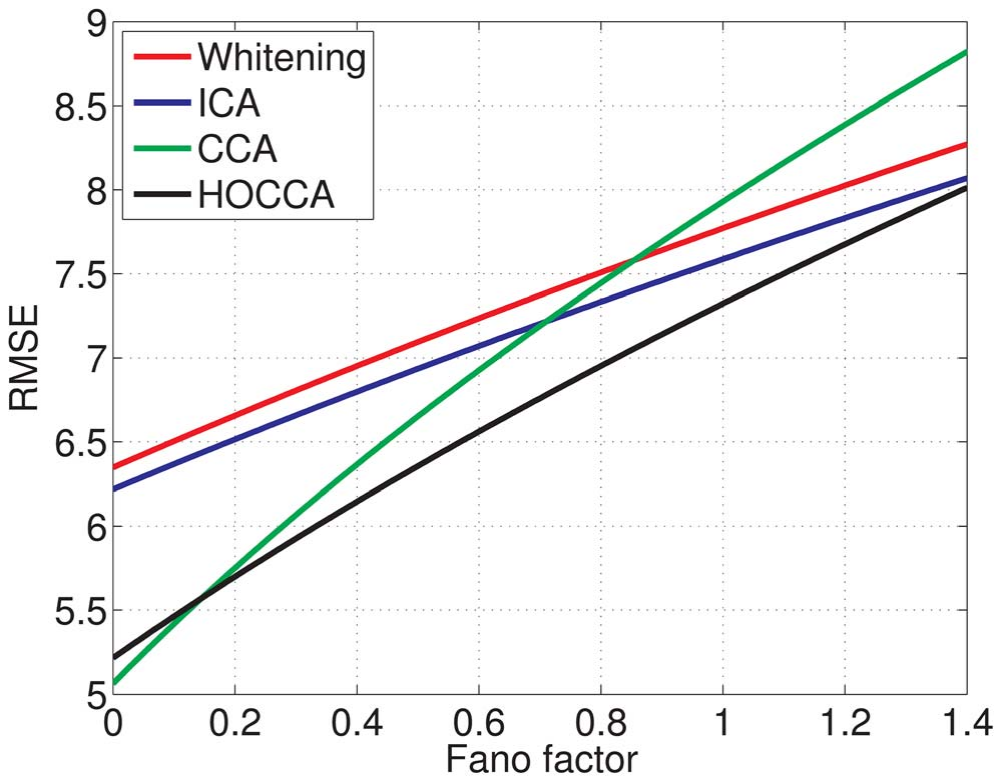
Noise-distortion curves. We compared the different methods in their ability to predict the representation of an image in daylight (CIE D65) from an image under yellowish light (CIE A) in the presence of neural noise. The curves show the root mean squared error (RMSE) of the predicted representation as the Fano factor (neural noise level) 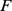 varies. The Fano factor in V1 is typically less than one, on average around 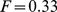 [32]. Representations which are sparse are less affected by the noise, representations which have a good correspondence give a low error for zero noise. HOCCA compares favorably to the other methods in the relevant regime of 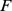 because it combines sparsity with good correspondence.

Figure 14 shows the root mean squared error (RMSE) of the prediction for the different methods as 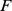 varies (noise-distortion curves). For 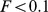, CCA gives the smallest error, which is reasonable because it minimizes the noise-free prediction error. For larger 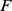, the importance of sparsity becomes visible. In a sparse representation, the introduced neural noise is lower on average. HOCCA has the smallest error from 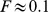 to 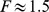 because it combines sparsity with good coupling. ICA is better than CCA for 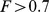, where the sparsity penalty starts to offset the noise-free prediction error. For 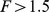, ICA yields the smallest error among all methods since its representation is the sparsest one. However, this regime of 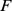 does not seem realistic for neurons in V1 [32]. Since the difference between CCA and HOCCA is rather small for 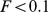, we conclude that HOCCA compares favorably to the other methods in the relevant regime of 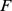: It combines good prediction accuracy with robustness to noise.

#### HOCCA in comparison to the alternative methods

We analyzed the learned representations of HOCCA, ICA, CCA, and whitening from both statistical and biological points of view using multiple criteria, see Table 1 for an overview. The different points of view yielded the same picture: While ICA performed well with regard to the individual representations (assessed by independence, sparsity, and plausibility of features), and CCA well with regard to correspondence (assessed by mutual information, similarity of features, and color psychophysics), HOCCA performed well in both aspects. The noise-distortion curves exemplified this favorable performance of HOCCA.

#### Predicting response-adaptation for spatio-chromatic inputs

The previous sections showed that HOCCA provides a single (unified) statistical framework to study both efficient representations and adaptation. In this section, we use HOCCA to make a testable prediction about the response of human spatio-chromatic sensors (neurons) to colored patterns under change of illumination.

HOCCA produced pairs of filters optimized for illumination CIE A and D65. Considering the two corresponding filters to be instances of the same (hypothetical) physical sensor when adapted to two different illuminants, we can investigate how adaptation changes the response to the same stimulus.

For the prediction, we used six representative HOCCA filter-pairs, three pairs with chromatic content in the red-green (RG) direction (feature-pairs 109, 134 and 152 with red frames in Figure 11) and three in the yellow-blue (YB) direction (feature-pairs 18, 67 and 78 with blue frames in Figure 11). With this choice, we consider filters of different spatial frequency and orientation for each chromatic content.

**Figure 15:**
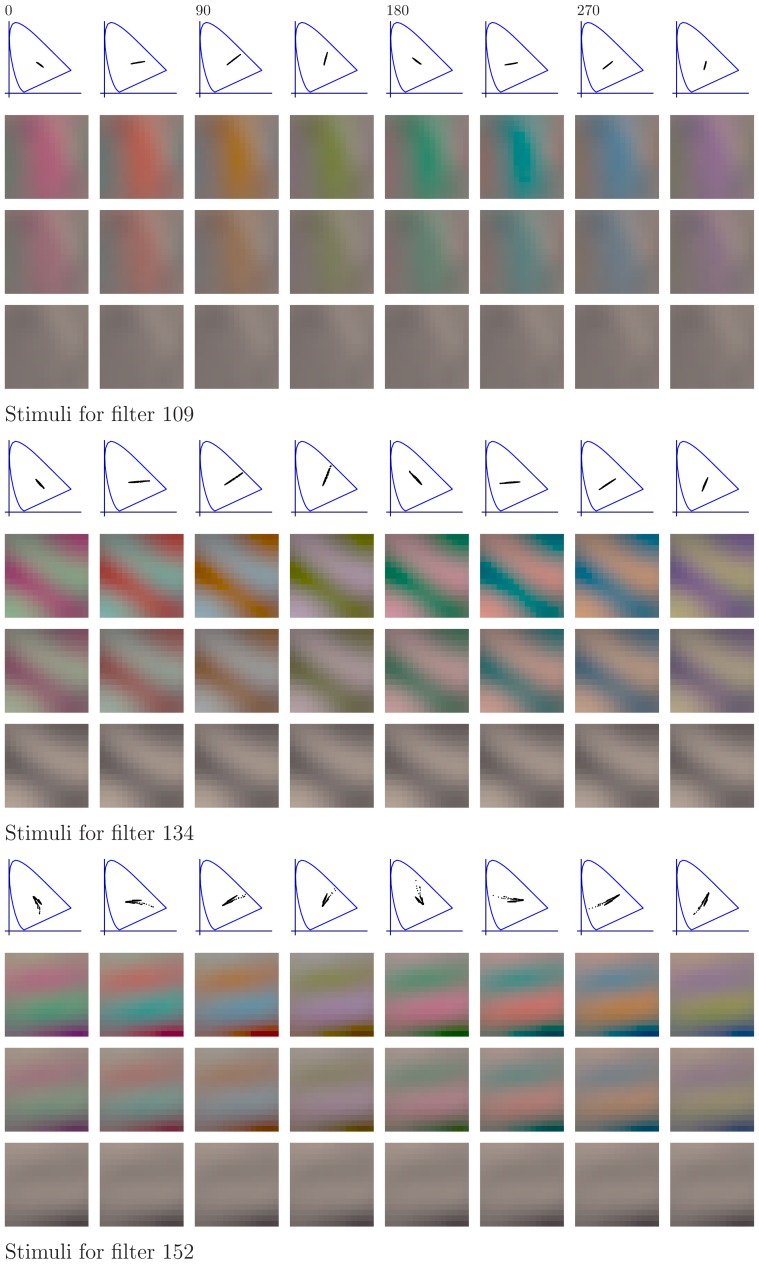
Stimuli used to predict the response-adaptation of RG sensors. Each 4-row panel corresponds to the stimuli used for one sensor. For each sensor, the stimuli were obtained by rotating and scaling the chromatic part of the optimal stimulus (the top left image in each panel). The color content changes in constant steps from left to right, and the scaling factor varies linearly from top to bottom, see Materials and Methods for details. The top row in each panel shows the chromatic diagrams for the first row of images.

**Figure 16:**
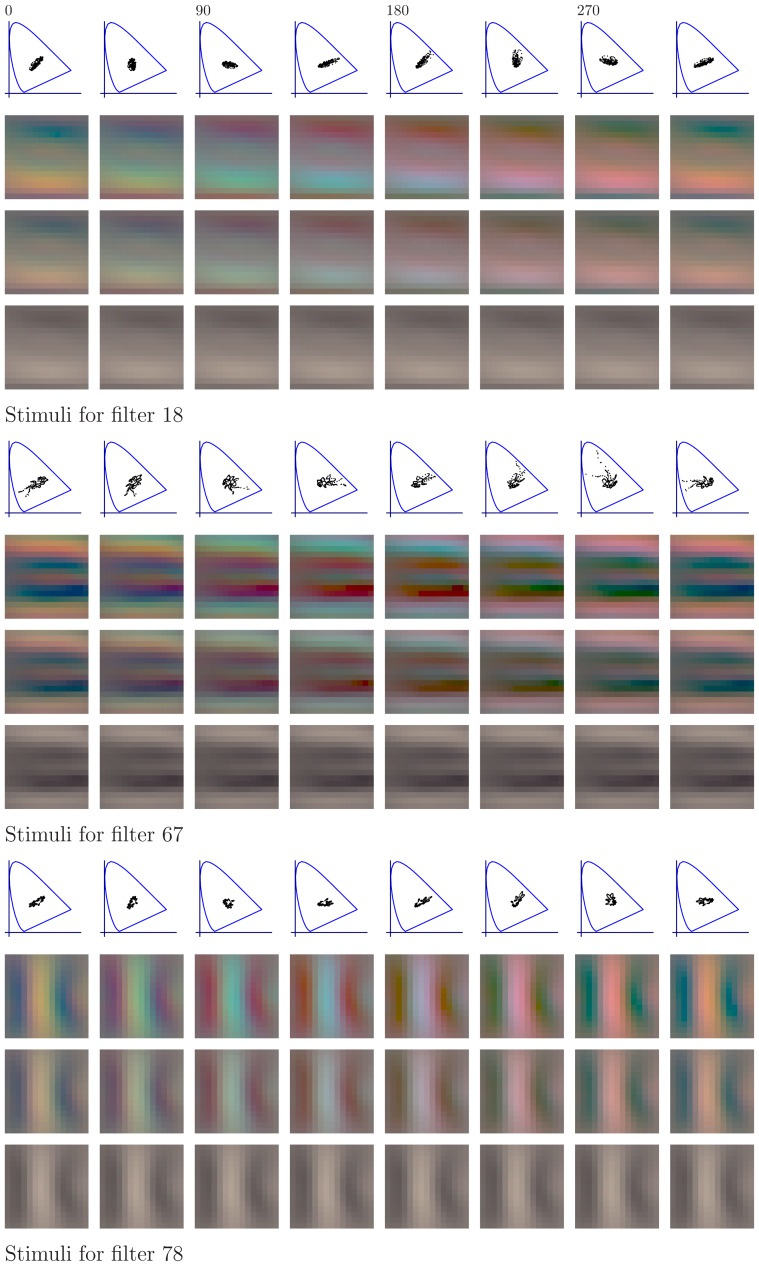
Stimuli used to predict the response-adaptation of YB sensors. The stimuli were generated as those in Figure 15.

For each sensor considered, we determined its optimal stimulus under illumination D65 and changed its chromatic contrast and its color content through rotations in the RG-YB plane, as done in [24] and [33], see Materials and Methods. Figures 15 and 16 show the obtained stimuli for the RG and YB filters, respectively. We used these stimuli both for the sensors adapted to illumination D65 and for the sensors adapted to illumination A. This allowed us to make a prediction of what should happen to the response to the same colored pattern when a (biological) sensor is adapted to illumination A instead of D65. To the best of our knowledge, there are no such measurements in the experimental literature.

**Figure 17:**
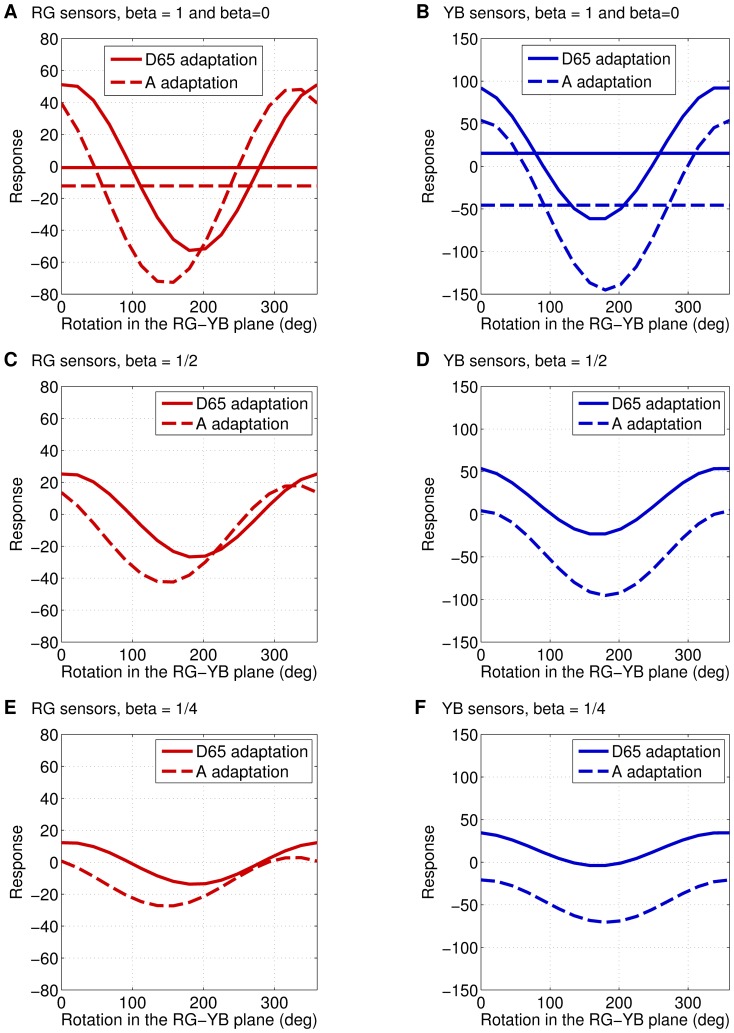
A testable prediction about response-adaptation for spatio-chromatic inputs. The figures show the average response of RG sensors and YB sensors when stimulated with the stimuli in Figures 15 and 16, respectively. Solid lines display responses of sensors adapted to CIE D65 illumination while dashed lines indicate adaptation to illumination CIE A. The constant curves in (a) and (b) are obtained for 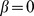. The optimal stimulus of a sensor adapted to illumination D65 is no longer optimal when the sensor is adapted to illumination A. We predict a shift in the response as adaptation to the new illumination occurs.

Figure 17 shows the average response of the considered RG and YB filters to the spatio-chromatic stimuli in Figures 15 and 16. Solid curves show the response of the sensor adapted to illumination D65, dashed curves the response when adapted to illumination A. HOCCA predicts sinusoidal oscillations as a function of the rotation angle of the chromatic modulation in the RG-YB plane. By definition of the stimuli used, the maximal responses are obtained for D65 illumination. The linear behavior implies linear reduction of the oscillation as the chromatic contrast decreases to zero. The response curves have an offset. This is due to the presence of an achromatic modulation in the stimuli, the filters learned by HOCCA are not purely chromatic. Interestingly, the solid and dashed curves do not have their optimum at the same angle. The optimal stimulus of a sensor adapted to illumination D65 is no longer optimal when the sensor is adapted to illumination A: We predict a shift in the responses as adaptation to the new illumination occurs.

## Discussion

We reported two sets of results in this paper. First, we proposed a new statistical method, called higher-order canonical correlation analysis (HOCCA), to jointly analyze multiple data sets. HOCCA combines desirable properties of canonical correlation analysis (CCA) and independent component analysis (ICA). HOCCA seeks independent and sparse sources inside each data set which have linear or variance correlations across the data sets. HOCCA is as widely applicable as CCA. Moreover, it generalizes CCA because it is not only sensitive to linear correlations but also to higher-order dependencies. We validated HOCCA on artificial data and proved that CCA emerges as a special case.

Second, we showed that HOCCA provides a single (unified) statistical framework to study visual processing under fixed lighting conditions and adaptation to new ones. Results on chromatic natural images demonstrated the benefits of jointly maximizing efficiency of representation and coupling across the data sets, as opposed to first maximizing efficiency and then finding a suitable coupling, or focusing on coupling only. We found that HOCCA features are consistent with the spatio-chromatic tuning properties of neurons in the primary visual cortex and that HOCCA reproduces corresponding colors psychophysics reasonably well. HOCCA provided us with a specific, experimentally testable prediction on how the response to colored patterns should change when the illumination changes.

### Relation to other statistical methods

We showed that HOCCA provides a generalization of CCA. CCA has been extended in many ways. Kernel CCA is a nonlinear extension of CCA that is sensitive to nonlinear dependencies across the data sets, see Section 3.2 of [34] and [35], [36]. One difference to our work is that kernel CCA does not yield an efficient representation of the data in terms of sparse canonical coordinates. Sparsity was incorporated in CCA [37], [38] but this was done on the level of the features and not on the level of the canonical coordinates as we do here.

CCA was also combined with ICA [39]. In that work, however, ICA serves more as a preprocessing step, and after the ICA rotation, the independent components are subject to a further rotation to maximize the nonlinear correlation across the data sets. In our context, such a rotation would, however, be suboptimal since rotating sparse independent components decreases their sparsity. In very recent work [40], the authors reversed the order of analysis (first analysis across the data sets, then finding independent sources within each data set). In our context, such an approach would, however, also be suboptimal since it does not seem to yield coupled canonical coordinates but only coupled subspaces.

In ICA, there is an ambiguity in the ordering of the column vectors of the mixing matrix. The joint estimation of 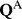 and 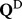 in HOCCA reduces this ambiguity because the ordering in both matrices must be the same: Due to the correspondence between canonical coordinates across the data sets, the ordering for one matrix cannot be changed without changing the ordering of the other in the same way.

In our simulations on natural images, we used a postprocessing stage after ICA where the mixing matrices 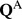 and 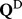 are re-ordered to obtain a correspondence. For natural image data, HOCCA was found to yield better results than this simple strategy. For other data, however, in particular if the individual data sets follow ICA models exactly, simple postprocessing of individual ICA results may work very well.

### Coupled representations learned from natural images

We applied HOCCA to two sets of images acquired with different illuminants, namely daylight-like CIE D65 and yellowish CIE A. HOCCA produced two sets of coupled filters, each one adapted to one of the illuminants. We compared HOCCA with three other statistical methods: Whitening by principal component analysis, ICA, and CCA. For HOCCA and CCA, the filters are learned together with their correspondence. For whitening and ICA, however, the filters are learned separately for each data set. We sought a correspondence after learning of the filters by finding pairs which had maximal mutual information.

Regarding the representations per data set, the mutual information reduction achieved by ICA and HOCCA is consistent with previously reported reductions for ICA in achromatic images [41], [42]. The filters learned by whitening and ICA are in line with previously reported results [13], [43], [44]. Further, our finding that ICA and HOCCA filters are less sensitive to chromatic than to achromatic gratings is consistent with sensitivity results in human vision [45].

Regarding correspondence, we found that, as HOCCA, CCA yielded a large amount of mutual information between the corresponding canonical coordinates even though CCA is only sensitive to linear correlations. The reason for this is two-fold: First, linear correlations contribute strongly to mutual information, see Figure 9, and CCA finds canonical coordinates which are maximally correlated. Second, even though CCA is only sensitive to linear correlations, this does not mean that the canonical coordinated obtained by CCA are Gaussian. In fact, Figure 7 shows that the marginals of the canonical coordinates of CCA are sparser than Gaussian random variables. This non-Gaussianity also contributes to mutual information.

Corresponding colors have been inferred from properties of natural images before [21]. The approach in the cited paper differs from the one in this paper in two main aspects: First, spatial information was not taken into account. Only the properties of the tristimulus pixel values were modeled. Second, the prediction of the corresponding colors was nonlinear. Compared to the linear methods used in this paper, nonlinear prediction is better suited to keep colors inside the chromatic diagram. Perceptually, this means that the nonlinear method avoids over-saturation of the predicted colors. Inspection of the predicted points in the chromatic diagram shows that the hues of the prediction, however, correspond better to the experimental data for HOCCA than for the nonlinear method.

In the joint learning of the features and the correspondence between them, HOCCA (and CCA) had access to the same images under two different illuminations. That is, the input data came labeled in terms of the illuminant (we used the superscripts ^A^ and ^D^ for the labeling). Furthermore, the objective function optimized in HOCCA consists of a sample average over several such observations. The visual system, however, is exposed to only one scene under one illumination at a time. While a sample average can be computed in an online fashion, assuming that the visual system has access to labeled input data is more problematic. However, information about the labels is often implicitly available and the labels can be inferred from it. For the inference of the labels, it is enough that the brain “knows” that an object under either of the two illuminations is the same (kind of) object. Such information about the identity of an object could be provided by top-down processes. For instance, when leaving a house with a red apple in the hand, the brain “knows” that the apple was not switched out but that the same object is in the hand both inside and outside, even though the radiance sensed by the eyes is different. This means that HOCCA should not be considered a mechanistic model of visual processing and adaptation; its neural implementation is left unspecified. Instead, HOCCA should be considered a normative theory based on statistical principles. It tells us what we can expect if efficiency and correspondence are optimized jointly, in case the same images under two different illuminations were available.

Adaptation to changes in illumination is related to illuminant compensation or color constancy. To compensate for the illuminant, additional measurements can be used, like measurements from a white object in the surround [27], [46], [47], or measurements from a wide ensemble of neighboring surfaces [48]–[51]. Another approach consists in mapping illuminant-dependent images to a domain which is illuminant independent [17]–[19], [21], [52]. The mappings can be seen as transforms which, like HOCCA, take into account the different statistical properties of the images in the different acquisition conditions.

HOCCA allowed us to make a testable prediction about the response to spatio-chromatic stimuli when adapted to CIE D65 or CIE A illumination. The prediction can be thought to correspond to the best-case scenario where labeled data has shaped the properties of the neurons. Next, we discuss the relation of our prediction to the experiments performed in [24] and [33]. In [24], responses to patterns with chromatic modulation in rotated directions of the red-green (RG), yellow-blue (YB) plane using a fixed white adaptation point similar to D65 were measured. The responses were found to oscillate sinusoidally as the stimulus rotated in the RG-YB plane. In [33], similar measurements were used to investigate the effect of habituation to high chromatic contrast stimuli modulated in certain directions of the color space. Again, a D65-like white average was used. In the control situation of zero contrast habituation stimuli, sinusoidal responses as in the aforementioned results were obtained. For non-zero habituation, these oscillations were found to shift and scale depending on the presence of linear or non-linear interactions between the basic RG-YB sensors.

Adaptation to illumination CIE D65 or CIE A is not exactly habituation to high chromatic contrast stimuli. Moreover, the linear nature of our filtering (computation of the canonical coordinates) cannot reproduce effects from eventual non-linear interactions. Therefore, our adaptation predictions cannot straightforwardly be compared to the habituation results reported in [33]. Nevertheless, we can notice interesting connections: First, both in our setup and in the aforementioned experimental work, smooth oscillations of the responses are obtained when the chromatic content of the optimal stimuli is changed, see Figure 16. Second, the offset of the curves is also similar to the reported experimental behavior. Third, the shifts in the responses which we predict as adaptation to the changed illumination occurs, are qualitatively similar to the shifts reported for contrast habituation.

## Materials and Methods

### Probabilistic generative model of HOCCA

We construct here HOCCA such that it takes higher-order statistical dependencies both within and across the data sets into account, in contrast to CCA. This allows us to find a both related and efficient representation of the data. The new method is based on a probabilistic generative model which we outline next. In Text S1, the model is generalized to the case where there are more than two data sets, each possibly of different dimensionality.

In order to find an efficient representation for each of the two data sets, we assume that each of the two vectors of canonical coordinates 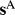 and 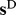 in (1) consists of statistically independent sparse random variables. The independence assumption concerns the elements within each vector only. In order to find features that are related across the data sets, we assume that the 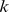-th random variable of 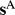 and the 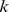-th random variable of 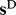 are statistically dependent.

The independence assumption for the canonical coordinates within a data set makes the whitened data 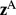 and 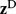 in (1) follow an ICA model with mixing matrices 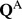 and 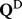. In this context, we call the canonical coordinates also sources.

Let 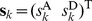 denote the column vector which contains the 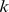-th canonical coordinate (source) from both data sets. With the above independence assumptions, the joint probability density function (pdf) of all the sources 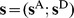 factorizes into 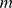 factors,
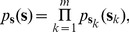
(8)where 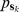 denotes the pdf of 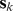. With the ICA models in (0) and the orthogonality of the mixing matrices, the joint pdf 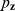 of the random variables 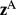 and 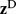 is
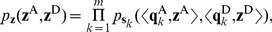
(9)where 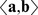 denotes the inner product between the two vectors 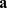 and 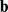. If 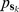 was known, 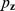 would be properly defined. We then could maximize the (rescaled) log-likelihood 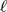,
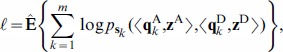
(10)to estimate the features 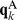 and 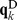, 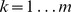. In the above equation, 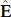 denotes the sample average over the joint observations of the whitened data sets 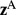 and 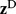.

While 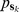 is generally unknown, we define it now such that we are capturing two possible types of dependencies between the data sets: linear correlation and variance dependencies. Linear correlation is presumably the simplest form of statistical dependency, and coupling in variance is the next simplest one. Variables which are linearly uncorrelated but correlated in variance tend to have high or low energies (squared values) at the same time. Modeling such dependencies proved useful when modeling the statistical dependencies within a given data set of natural images, see Chapter 10 of [2].

Sources 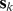 with linear and variance dependencies can be generated via
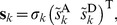
(11)where 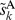 and 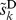 are two zero mean Gaussian random variables with correlation coefficient 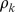, and 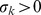 is the variance variable responsible for the scaling. We prove in Text S1, Section S1.1, that the distribution 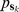 has the form
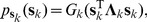
(12)where 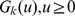 is a monotonically decreasing, strictly convex function which depends on the prior for 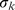 and the correlation coefficient 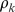. It is further shown that the same also holds for 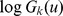. Direct calculations, or the derivation in the supporting text, show that the correlation coefficient between 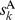 and 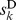 is given by 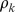. The matrix 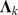 is the precision matrix (inverse covariance matrix) of 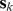. Since the sources in ICA are commonly assumed to have variance one, 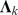 is given by (3).

While different choices are possible for 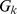, an interesting family of functions is obtained by assuming that 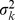 follows an inverse Gamma distribution. As shown in Text S1, Section S1.2, the functions 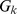 are then given by 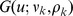 in (4). The resulting pdf 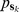 is bivariate student's t. The HOCCA objective function in (2) follows from (10) with this choice for 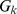.

The family 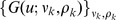 is interesting since the shape parameter 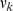 controls the extent of higher-order statistical dependencies between 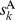 and 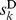 while the correlation coefficient 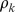 captures their linear correlation. This can best be seen by considering the mutual information between 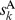 and 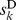. Mutual information measures the amount of information about 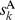 that one can obtain from 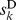, and vice versa [53], see (S1–34) in Text S1, Section S1.2, for the formal definition. For the bivariate student's t distribution 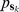, the mutual information 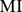 consists of two parts [54],
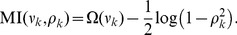
(13)The analytical expression for the first part, 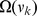, is given in (S1–36) in Text S1. The function 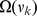 decreases to zero as 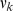 increases. The second part depends only on 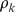 and corresponds to the mutual information between two Gaussian random variables with correlation coefficient 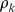. Hence, for large 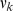 when 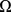 becomes small, the correlation coefficient 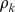 captures already most of the dependency between 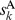 and 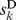. If 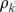 goes to zero and 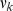 is large, 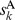 and 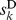 become statistically independent. If 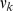 is small, on the other hand, there are higher-order statistical dependencies. Figure 8 shows the non-Gaussian part 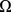 and the Gaussian part as a function of 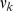 and 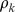, respectively. The figure shows that a value of 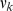 close to two contributes to the mutual information like a correlation coefficient 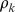 of about 0.65, 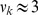 corresponds to 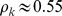. Furthermore, the shape parameter 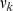 affects the non-Gaussianity (sparsity) of the marginal distributions of 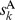 and 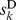: The marginal distributions are univariate student's t distributions with the same shape parameters 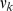 as 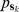 [55]. As 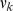 decreases, the distributions become more heavy-tailed and peaked around zero, that is, the random variables are more sparse.

### Simulations on artificial data

We used artificial data to validate HOCCA. We give here details for the data generation and the performance measures used in the assessment and in the comparison with CCA.

The data was generated according to (1). The dimensionality was 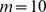 and the mixing matrices 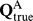 and 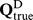 were randomly generated by independently drawing the elements of the matrices from a standard normal distribution, followed by orthonormalization of each matrix. The correlation coefficients 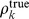 between 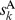 and 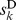 were drawn from an uniform distribution on (−1 −0.1
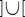
0.1 1), and the parameters 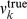 from an uniform distribution on 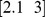. The true canonical coordinates were thus sparse and linearly correlated. We avoided sampling correlation coefficients close to zero since CCA is sensitive to linear correlation only.

We analyzed the estimation results of HOCCA and CCA using three measures of performance. The first measure assesses how well the mixing matrices (features) were recovered, the second the efficiency of the learned representation, and the third how well the coupling (correspondence) between the two data sets was identified. We assessed the efficiency of the representation from a sparsity and a related information theoretical point of view. Note that the first two measures are insensitive to the coupling between the data sets.

In order to quantify the accuracy of the estimated matrices 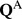 and 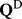, we used the Amari index 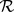 [56],
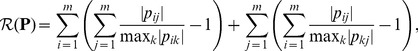
(14)applied to 
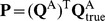
 and 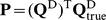. The entry in row 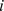 and column 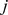 of the matrix 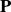 is denoted by 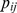. The index is zero if 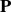 is a permutation matrix. For 10 dimensional random matrices formed by independent standard normal random variables, the index takes typically values around 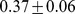 (average ± two standard deviations).

In order to quantify the sparsity of the recovered canonical coordinates 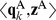 and 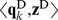, we used the index [57]

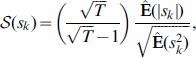
(15)applied to 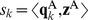 and 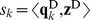, after removal of their mean. As before, 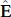 denotes the sample average and we took 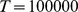 data points to compute it. The index 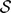 is non-decreasing with increasing sparsity; it takes zero as minimal and one as maximal value. A Gaussian has a value of 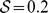.

In order to measure the efficiency of the learned representation from an information theoretical point of view, we computed by which extent the mutual information between the recovered coordinates was smaller than the mutual information between the original (white) data. This difference in mutual information is called multi-information reduction. We can here compute it by comparing the entropies of the marginal pdfs of the (white) data and the recovered canonical coordinates [41]. In our context, multi-information reduction is related to sparsity maximization since sparse variables have a smaller entropy than Gaussian variables of the same variance. We computed the multi-information reduction using 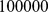 data points.

In order to assess how well the coupling between the two data sets was identified, we computed the mutual information between the inferred corresponding sources 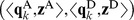, and compared it to the mutual information of the “true” corresponding sources 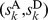. We measured mutual information using the maximum likelihood estimator with Miller-Maddow correction [58]. For computation of the mutual information, we used five million data points, and 1000 bins for the joint histogram after uniformization of the marginals.

### Natural image data and preprocessing

The data used for the learning consists of pairs of images (image patches) that we extracted from a set of 50 larger natural images acquired under CIE D65, daylight, and under CIE A, yellowish light. Figure 2 shows pairs of example images from the database. The database is publicly available at http://isp.uv.es/data_color.htm and a detailed description was given before [21]. The images of the database are given in standard CIE XYZ tristimulus values. This makes it an appropriate data set to reproduce classical psychophysical results since they were obtained with these standard illuminants.

We used 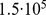 corresponding image patches of size 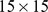 pixels which we extracted from the pairs of larger images at the same randomly chosen position. After removal of the mean, the patches from images taken under illumination D65 give 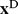, and the patches from images under illumination A are 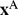. Each pair of images 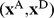 shows the same extract of the larger visual scene under two different illuminants. The dimension of 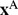 and 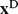 is 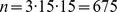.

**Figure 18:**
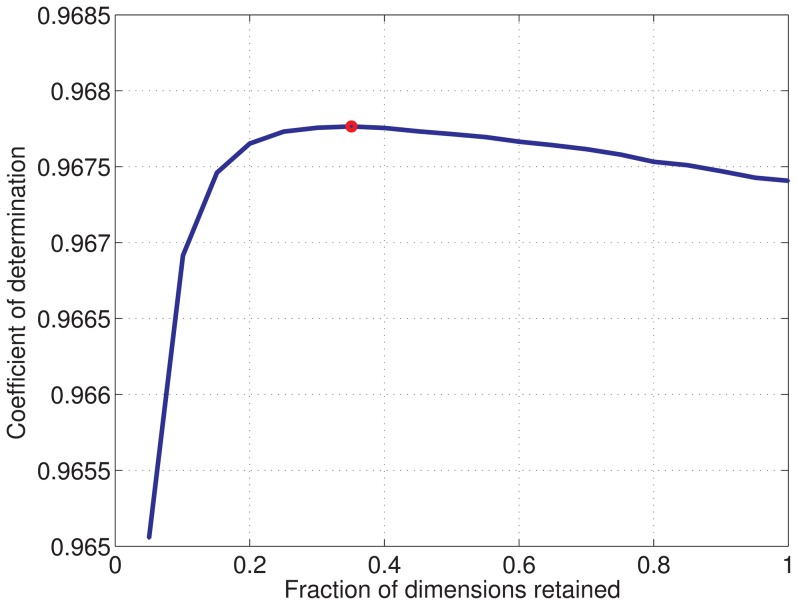
Choosing the amount of dimension reduction based on the performance when 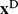 is linearly predicted from 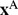. The plot shows the coefficient of determination (fraction of explained variance) as a function of the retained dimension 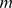 of 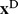 and 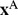. Retaining about 35% of the dimensions (236 out of 675) gives the best performance. Retaining 236 dimensions removes 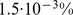 and 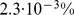 of the total variance of 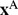 and 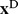, respectively.

We then performed whitening and reduced the dimensionality of each individual data set by principal component analysis (PCA). Dimension reduction is worthwhile if there are strong correlations in the data, that is, if the data is essentially located in a subspace of lower dimensionality than 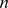. Reducing the dimension of 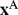 and 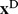 can then reduce the average prediction error when trying to linearly predict 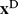 from 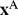, or vice versa (see Text S2, Section S2.2). In order to objectively decide about the amount of dimension reduction, we used the fraction of variance accounted for by the prediction (coefficient of determination 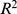) when image patches under D65 illumination are linearly predicted from PCA truncated patches under illumination A. Figure 18 shows the coefficient of determination as a function of the retained dimension 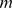 of the data. According to the behavior in Figure 18 we decided to reduce the dimension of 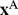 and 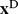 from 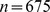 to 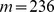. Retaining 236 dimensions removes only 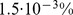 and 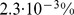 of the variance of 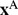 and 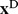, respectively.

### Learning representations of natural images

We used HOCCA to learn the coupled representation by maximizing the objective 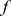 in (2). Other statistical methods can also be used to learn coupled representations, that is, the matrices 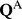 and 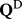 in Figure 3. We compared HOCCA to three alternative methods: canonical correlation analysis (CCA), a method based on whitening by principal component analysis, and a method based on independent component analysis (ICA). Table 1 provides an overview of the methods used.

CCA is briefly reviewed in Text S2. HOCCA and CCA naturally lead to coupled canonical representations. Whitening and ICA, however, are specific to each data set itself. After initial whitening or ICA, separately performed on each data set, we thus matched the learned filters across the data sets by greedily choosing pairs of components which had maximal mutual information. In this way, we obtained a coupled representation that can be used in the comparison with HOCCA.

Comparing HOCCA with the whitening-based approach is interesting since whitening is the first step in all methods. Comparing HOCCA with CCA and the ICA-based approach is interesting since these methods can be considered to represent limiting cases of HOCCA: ICA features are obtained by maximizing the efficiency (sparsity) of the representation of each data set individually, without concern for a possible correspondence between them. CCA features are obtained by maximization of correspondence (measured by linear correlation), without concern for the efficiency (sparsity) of the individual representations. HOCCA, on the other hand, is jointly maximizing the efficiency of the individual representations and the correspondence between them.

### Analyzing the learned representations of natural images

The representations were statistically analyzed by assessing their efficiency and the coupling between the corresponding canonical coordinates (feature outputs) 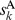 and 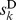. Efficiency was measured using multi-information reduction and sparsity, coupling was measured using mutual information. These measurements were performed as in the analysis of the results on artificial data.

For the visualization of the learned features, the features were first scaled to have unit norm and then contrast-normalized by applying a global scaling factor to the deviation from the average. The scaling was chosen so that the feature colors are reproducible in conventional displays: Too small scaling factors lead to chromatically uniform features while too large factors give rise to non-reproducible imaginary colors, that is, to negative luminance or to colors outside the reproducible gamut.

We reproduced corresponding-colors based on the learned representations as follows: Given a spatially uniform patch of a certain color under illumination CIE A, we identified it with 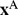 in Figure 2 and represented it using the canonical coordinates 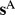. Then, we predicted the 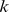-th canonical coordinates 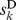 from 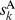, and transformed back to the original pixel-representation, that is, to 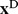, which yielded the corresponding color under illumination CIE D65. Given colors under illumination D65, the procedure was reversed. The prediction of 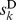 from 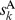 (and vice versa) was constrained to be linear, even though nonlinear prediction would be possible too. In more detail, since the canonical coordinates have zero mean and unit variance, the prediction of 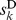 is 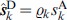, where 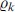 is the correlation coefficient between 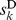 and 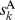.

For the noise-distortion curves, the setup of the corresponding-colors was modified in two aspects: First, we used image data with spatial structure. Second, the internal representation by means of the canonical coordinates was subject to noise. The noisy version of 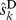 is denoted by 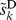,
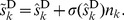
(16)The random variables 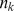 are independent from each other and from the canonical coordinates, and have zero mean and unit variance. For a fixed image 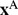, the noisy response 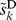 fluctuates around the mean 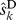 with a variance 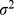. The variance was assumed to be signal dependent,
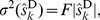
(17)where 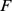 is the Fano factor (index of dispersion) of the noisy response. We measured to which extent the noisy inferred representation deviates from the noise-free representation of the same image under illumination D65. That is, we measured how much 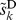 deviates from 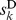. We used the root mean squared error (RMSE), 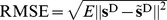 as error metric. The analytical expression for the squared error reported in (7) is derived in Text S3.

For the stimuli used in the prediction, we changed the chromatic contrast and color content as follows: In a achromatic red-green yellow-blue representation, the color 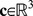 of each pixel can be seen as an achromatic and a chromatic departure from the average 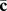: 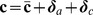. The average can further be divided into an achromatic (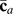) and chromatic part (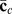). The stimuli were obtained through rotations of 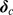, via a 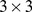 rotation matrix 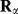, and by scaling the resulting chromatic part: 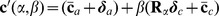. The color content was rotated in constant steps, and the scaling factor 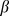 was varied linearly from one to zero.

## Supporting Information

Text S1 **Details of Higher-Order Canonical Correlation Analysis.**
(PDF)

Text S2 **Background Material from Multivariate Analysis.**
(PDF)

Text S3 **Calculations for the Noise-Distortion Analysis.**
(PDF)
