# Supplementary material for: Spatio-Chromatic Adaptation via Higher-Order Canonical Correlation Analysis of Natural Images
Source: PLoS One. 2014 Feb 12;9(2):e86481. doi: 10.1371/journal.pone.0086481 (PMC3922757; doi:10.1371/journal.pone.0086481)
Supplement: Text S1 — Details of Higher-Order Canonical Correlation Analysis. (PDF) [file pone.0086481.s001.pdf]

S1 Details of Higher-Order Canonical Correlation Analysis

We present here the mathematical details of higher-order canonical correlation analysis (HOCCA). Section S1.1 deals with the (general) probabilistic data model that underlies HOCCA. In Section S1.2, we insert more assumptions and derive the parametric formulation presented in the main text. Section S1.3 contains the details on the relation to canonical correlation analysis.

S1.1 General nonparametric case

We consider here the generalized case of n_c coupled data sets of possibly different dimensionality. Equation (1) generalizes to

$$\mathbf{z}^i = \mathbf{Q}^i \mathbf{s}^i \quad (i = 1, \ldots, n_c), \tag{S1-1}$$

where $\mathbf{z}^i, \mathbf{s}^i \in \mathbb{R}^{m_i}$. We assume that the first $m = \min(m_1, \ldots, m_{n_c})$ canonical coordinates of the data sets are possibly coupled with each other while the remaining coordinates are independent. Thus, the joint pdf of the sources decomposes as

$$p_{\mathbf{s}}(s_1^1, \ldots, s_{m_{n_c}}^{n_c}) = \prod_{k=1}^{m} p_{\mathbf{s}_k}(\mathbf{s}_k) \prod_{i=1}^{n_c} \prod_{k=m+1}^{m_i} p_{ik}(s_k^i) \tag{S1-2}$$

where the m vectors $\mathbf{s}_k$,

$$\mathbf{s}_k = (s_k^1, \ldots, s_k^{n_c})^\top, \tag{S1-3}$$

contain the possibly coupled coordinates, and the p_{ik} are the pdfs of the non-coupled sources.

We next specify $p_{\mathbf{s}_k}$. We assume that the elements s_k^i of the vector $\mathbf{s}_k$ are coupled via

$$s_k^1 = \sigma_k \tilde{s}_k^1, \qquad s_k^2 = \sigma_k \tilde{s}_k^2, \qquad s_k^3 = \sigma_k \tilde{s}_k^3, \qquad \ldots \qquad s_k^{n_c} = \sigma_k \tilde{s}_k^{n_c}, \tag{S1-4}$$

where the random variable $\sigma_k > 0$ sets the variance, and the $\tilde{s}_k^i$ are zero mean Gaussian random variables. The distribution of σ_k affects the strength of the coupling. Inverting the linear transform in (S1-4) gives

$$\tilde{\mathbf{s}}_k = \frac{1}{\sigma_k} \mathbf{s}_k, \tag{S1-5}$$

where $\tilde{\mathbf{s}}_k = (\tilde{s}_k^1, \ldots, \tilde{s}_k^{n_c})$. The determinant of this linear transformation is $1/\sigma_k^{n_c}$. Integrating out the variable σ_k with density p_{σ_k} leads to an expression for the density of $\mathbf{s}_k$,

$$p_{\mathbf{s}_k}(\mathbf{s}_k) = \int \frac{p_{\sigma_k}(\sigma_k)}{\sigma_k^{n_c}} p_{\tilde{\mathbf{s}}_k}\left(\frac{\mathbf{s}_k}{\sigma_k}\right) \mathrm{d}\sigma_k. \tag{S1-6}$$

Equivalently, we can specify a prior p_{ω_k} for $\omega_k = \sigma_k^2$. The density $p_{\mathbf{s}_k}$ is then

$$p_{\mathbf{s}_k}(\mathbf{s}_k) = \int \frac{p_{\omega_k}(\omega_k)}{\omega_k^{n_c/2}} p_{\tilde{\mathbf{s}}_k}\left(\frac{\mathbf{s}_k}{\sqrt{\omega_k}}\right) \mathrm{d}\omega_k. \tag{S1-7}$$

The variables $\tilde{\mathbf{s}}_k$ are assumed jointly Gaussian with density $p_{\tilde{\mathbf{s}}_k}$,

$$p_{\tilde{\mathbf{s}}_k}(\tilde{\mathbf{s}}_k) = \frac{1}{(2\pi)^{\frac{n_c}{2}} |\tilde{\mathbf{\Sigma}}_k|^{\frac{1}{2}}} \exp\left(-\frac{1}{2} \tilde{\mathbf{s}}_k^\top \tilde{\mathbf{\Sigma}}_k^{-1} \tilde{\mathbf{s}}_k\right), \tag{S1-8}$$

where $\tilde{\mathbf{\Sigma}}_k$ is the covariance matrix. The covariance matrix $\mathrm{V}(\mathbf{s}_k)$ of $\mathbf{s}_k$ is

$$\begin{aligned} \mathrm{V}(\mathbf{s}_k) &= \int_0^\infty p_{\omega_k}(\omega_k)\, \mathrm{V}(\mathbf{s}_k|\omega_k) \mathrm{d}\omega_k && \text{(S1-9)} \\ &= \mathrm{V}(\tilde{\mathbf{s}}_k) \int_0^\infty \omega_k p_{\omega_k}(\omega_k) \mathrm{d}\omega_k && \text{(S1-10)} \\ &= \tilde{\mathbf{\Sigma}}_k \mu_k, && \text{(S1-11)} \end{aligned}$$

where μ_k denotes the mean of ω_k. For the second equality, we have used that $\mathrm{V}(\mathbf{s}_k|\omega_k) = \omega_k \, \mathrm{V}(\tilde{\mathbf{s}}_k)$. The covariance matrix $\mathrm{V}(\mathbf{s}_k)$ is proportional to $\tilde{\mathbf{\Sigma}}_k$, which means that the correlation coefficient between s_k^i and s_k^j is the same as the correlation coefficient between $\tilde{s}_k^i$ and $\tilde{s}_k^j$, $i \neq j$. Further,

$$\tilde{\mathbf{\Sigma}}_k^{-1} = \mu_k \mathbf{\Lambda}_k, \tag{S1-12}$$

where $\mathbf{\Lambda}_k = \mathrm{V}(\mathbf{s}_k)^{-1}$ is the precision matrix of $\mathbf{s}_k$. Hence, the prior $p_{\mathbf{s}_k}$ is

$$p_{\mathbf{s}_k}(\mathbf{s}_k) = G_k(\mathbf{s}_k^\top \mathbf{\Lambda}_k \mathbf{s}_k), \tag{S1-13}$$

where the function G_k is defined via the one-dimensional integral

$$G_k(u) = \frac{1}{(2\pi)^{\frac{n_c}{2}} |\tilde{\mathbf{\Sigma}}_k|^{\frac{1}{2}}} \int_0^\infty \exp\left(-\frac{\mu_k}{2\omega_k} u\right) \frac{p_{\omega_k}(\omega_k)}{\omega_k^{n_c/2}} \mathrm{d}\omega_k \quad (u \geq 0), \tag{S1-14}$$

which depends on the prior p_{ω_k} and the covariance matrix of $\tilde{\mathbf{s}}_k$. Taking the derivative under the integral sign, and using that $\mu_k > 0$, we find that that $G_k'(u) < 0$. Taking the second derivative shows further that $G_k''(u) > 0$. Hence, G_k is monotonically decreasing and strictly convex for $u > 0$. The same also holds for $\log G_k$: $(\log G_k(u))' = G_k'(u)/G_k(u) < 0$ since G_k is positive, and $(\log G_k(u))'' > 0$ follows from a development as in Section 10.8 of [2] using the Cauchy-Schwarz inequality.

By orthogonality of $\mathbf{Q}^i$, the joint distribution of $\mathbf{z} = (\mathbf{z}^1, \ldots, \mathbf{z}^{n_c})^\top$ is

$$\begin{aligned} p_{\mathbf{z}}(\mathbf{z}^1, \ldots, \mathbf{z}^{n_c}) &= p_{\mathbf{s}}(\langle \mathbf{q}_1^1, \mathbf{z}^1 \rangle, \ldots, \langle \mathbf{q}_{m_{n_c}}^{n_c}, \mathbf{z}^{n_c} \rangle) &\text{(S1-15)} \\ &= \prod_{k=1}^m p_{\mathbf{s}_k}(\langle \mathbf{q}_k^1, \mathbf{z}^1 \rangle, \ldots, \langle \mathbf{q}_k^{n_c}, \mathbf{z}^{n_c} \rangle) \prod_{i=1}^{n_c} \prod_{k=m+1}^{m_i} p_{ik}(\langle \mathbf{q}_k^i, \mathbf{z}^i \rangle). &\text{(S1-16)} \end{aligned}$$

Denoting the n_c-dimensional vector $(\langle \mathbf{q}_k^1, \mathbf{z}^1 \rangle, \ldots, \langle \mathbf{q}_k^{n_c}, \mathbf{z}^{n_c} \rangle)^\top$ by $\mathbf{y}_k$, the t-th observation of $\mathbf{z}^i$ by $\mathbf{z}^i(t)$, and the t-th observation of $\mathbf{y}_k$ by $\mathbf{y}_k(t)$, we obtain the log-likelihood ℓ,

$$\ell = \sum_{t=1}^T \sum_{k=1}^m \log G_k(\mathbf{y}_k(t)^\top \mathbf{\Lambda}_k \mathbf{y}_k(t)) + \sum_{t=1}^T \sum_{i=1}^{n_c} \sum_{k=m+1}^{m_i} \log p_{ik}(\langle \mathbf{q}_k^i, \mathbf{z}^i(t) \rangle). \tag{S1-17}$$

Here, T denotes the total number of observations and we tacitly assume that we can easily evaluate G_k and p_{ik}.

The log-likelihood separates into two parts: The first part with the G_k contains the possibly coupled features while the second part with the p_{ik} contains the remaining ones. The two parts are independent from each other up to the orthogonality constraint that $\langle \mathbf{q}_k^i, \mathbf{q}_j^i \rangle = 0$ for $k \neq j$. Moreover, the second part separates into n_c independent sub-parts. Hence, to maximize the log-likelihood, it is possible to maximize f,

$$\begin{aligned} f &= \frac{1}{T} \sum_{k=1}^m \log G_k(\mathbf{y}_k(t)^\top \mathbf{\Lambda}_k \mathbf{y}_k(t)) &\text{(S1-18)} \\ &= \sum_{k=1}^m \hat{\mathrm{E}} \log G_k(\mathbf{y}_k^\top \mathbf{\Lambda}_k \mathbf{y}_k), &\text{(S1-19)} \end{aligned}$$

in a first step, and afterwards the remaining terms J_i,

$$\begin{aligned} J_i &= \frac{1}{T} \sum_{t=1}^T \sum_{k=m+1}^{m_i} \log p_{ik}(\langle \mathbf{q}_k^i, \mathbf{z}^i(t) \rangle) &\text{(S1-20)} \\ &= \sum_{k=m+1}^{m_i} \hat{\mathrm{E}} \log p_{ik}(\langle \mathbf{q}_k^i, \mathbf{z}^i \rangle) &\text{(S1-21)} \end{aligned}$$

for $i = 1 \dots n_c$. In the equations, the symbol $\hat{\mathrm{E}}$ denotes the sample average. Optimizing the terms J_i corresponds to doing ordinary ICA on the individual $\mathbf{z}^i$ under the constraint that the $\mathbf{q}_k^i$, $k = m{+}1 \dots m_i$, are orthogonal to the $\mathbf{q}_k^i$, $k = 1 \dots m$, which are obtained in the optimization of f. If we are interested in the possibly coupled features only, it suffices to maximize f.

S1.2 Convenient parametrization

We derive here a convenient family of functions for the G_k. We consider the case where the variance variable $\omega_k = \sigma_k^2$ follows the inverse Gamma distribution with parameters $\alpha_k > 1$, $\beta_k > 0$,

$$p_{\omega_k}(\omega_k; \alpha_k, \beta_k) = \frac{\beta_k^{\alpha_k}}{\Gamma(\alpha_k)} \omega_k^{-\alpha_k - 1} \exp\left(-\frac{\beta_k}{\omega_k}\right). \tag{S1-22}$$

Here, $\Gamma(\alpha_k)$ is the gamma function,

$$\Gamma(\alpha_k) = \int_0^\infty u^{\alpha_k - 1} \exp(-u) \mathrm{d}u. \tag{S1-23}$$

The mean μ_k of ω_k is $\beta_k/(\alpha_k - 1)$. The function $G_k(u)$ in (S1-14) becomes thus

$$G_k(u) = \frac{\beta_k^{\alpha_k}}{\Gamma(\alpha_k)} \frac{1}{(2\pi)^{\frac{n_c}{2}} |\tilde{\mathbf{\Sigma}}_k|^{\frac{1}{2}}} \int_0^\infty \omega_k^{-\alpha_k - 1 - \frac{n_c}{2}} \exp\left(-\left(\beta_k + \frac{\beta_k}{2(\alpha_k - 1)} u\right) \frac{1}{\omega_k}\right) \mathrm{d}\omega_k. \tag{S1-24}$$

Making the change of variables

$$\omega_k = \left(\beta_k + \frac{\beta_k}{2(\alpha_k - 1)} u\right) \frac{1}{v} \tag{S1-25}$$

we obtain

$$\begin{aligned} G_k(u) &= \frac{\beta_k^{\alpha_k}}{\Gamma(\alpha_k)} \frac{1}{(2\pi)^{\frac{n_c}{2}} |\tilde{\mathbf{\Sigma}}_k|^{\frac{1}{2}}} \int_0^\infty \left(\beta_k + \frac{\beta_k}{2(\alpha_k - 1)} u\right)^{-\alpha_k - \frac{n_c}{2}} v^{\alpha_k + \frac{n_c}{2} - 1} \exp(-v) \, \mathrm{d}v \\ &= \frac{\beta_k^{\alpha_k}}{\Gamma(\alpha_k)} \frac{1}{(2\pi)^{\frac{n_c}{2}} |\tilde{\mathbf{\Sigma}}_k|^{\frac{1}{2}}} \left(\beta_k + \frac{\beta_k}{2(\alpha_k - 1)} u\right)^{-\alpha_k - \frac{n_c}{2}} \Gamma\left(\alpha_k + \frac{n_c}{2}\right) \qquad \text{(S1-26)} \\ &= \frac{\Gamma\left(\alpha_k + \frac{n_c}{2}\right)}{\Gamma(\alpha_k)} \frac{1}{(2\pi\beta_k)^{\frac{n_c}{2}} |\tilde{\mathbf{\Sigma}}_k|^{\frac{1}{2}}} \left(1 + \frac{1}{2(\alpha_k - 1)} u\right)^{-\alpha_k - \frac{n_c}{2}}. \qquad \text{(S1-27)} \end{aligned}$$

From (S1-12), we have

$$|\tilde{\mathbf{\Sigma}}_k|^{-\frac{1}{2}} = \mu_k^{\frac{n_c}{2}} |\mathbf{\Lambda}_k|^{\frac{1}{2}}, \tag{S1-28}$$

and as $\mu_k = \beta_k/(\alpha_k - 1)$, we obtain

$$|\tilde{\mathbf{\Sigma}}_k|^{-\frac{1}{2}} = \left(\frac{\beta_k}{\alpha_k - 1}\right)^{\frac{n_c}{2}} |\mathbf{\Lambda}_k|^{\frac{1}{2}}, \tag{S1-29}$$

so that

$$G_k(u) = \frac{\Gamma\left(\alpha_k + \frac{n_c}{2}\right)}{\Gamma(\alpha_k)} \frac{1}{(2\pi(\alpha_k - 1))^{\frac{n_c}{2}}} |\mathbf{\Lambda}_k|^{\frac{1}{2}} \left(1 + \frac{1}{2(\alpha_k - 1)} u\right)^{-\alpha_k - \frac{n_c}{2}}, \tag{S1-30}$$

which does not depend on the parameter β_k. Introducing the parameter $\nu_k = 2\alpha_k > 2$, the function $G_k(u)$ is

$$G_k(u) = \frac{\Gamma\left(\frac{\nu_k + n_c}{2}\right)}{\Gamma\left(\frac{\nu_k}{2}\right)} \frac{|\mathbf{\Lambda}_k|^{\frac{1}{2}}}{(\pi(\nu_k - 2))^{\frac{n_c}{2}}} \left(1 + \frac{u}{\nu_k - 2}\right)^{-\frac{\nu_k + n_c}{2}}, \tag{S1-31}$$

which is (4) in the main text of the paper for $n_c = 2$.

The random vector $\mathbf{s}_k$ has the density $p_{\mathbf{s}_k}(\mathbf{s}_k) = G_k(\mathbf{s}_k^\top \mathbf{\Lambda}_k \mathbf{s}_k)$, see (S1-13). The re-scaled random vector $\mathbf{t}$,

$$\mathbf{t} = \mathbf{s}_k \sqrt{\frac{\nu_k}{\nu_k - 2}}, \tag{S1-32}$$

has the density $p_{\mathbf{t}}$,

$$p_{\mathbf{t}}(\mathbf{t}) = \frac{\Gamma\left(\frac{\nu_k + n_c}{2}\right)}{\Gamma\left(\frac{\nu_k}{2}\right)} \frac{|\mathbf{\Lambda}_k|^{\frac{1}{2}}}{(\pi \nu_k)^{\frac{n_c}{2}}} \left(1 + \frac{\mathbf{t}^\top \mathbf{\Lambda}_k \mathbf{t}}{\nu_k}\right)^{-\frac{\nu_k + n_c}{2}}, \tag{S1-33}$$

which is the parametrization of a n_c-variate student's t distribution.

Mutual information MI between n random variables $y_1, \ldots, y_n$ is defined as the Kullback Leibler divergence between their joint pdf $p(y_1, \ldots, y_n)$ and the product of their marginal pdfs $\prod_i p(y_i)$,

$$\mathrm{MI} = \int p(y_1, \ldots, y_n) \log \frac{p(y_1, \ldots, y_n)}{\prod_i p(y_i)} \mathrm{d}y_1 \ldots \mathrm{d}y_n. \tag{S1-34}$$

Mutual information between several random variables is also known as multi-information [41]. For the n_c-variate student's t distribution, the mutual information MI is [54]

$$\mathrm{MI} = \Omega(\nu_k) + \frac{1}{2} \log |\mathbf{\Lambda}_k|, \tag{S1-35}$$

where

$$\begin{aligned} \Omega(\nu) \quad = \quad & \log \left[\frac{\Gamma\left(\frac{n_c}{2}\right)}{\pi^{\frac{n_c}{2}}} \frac{\left(\beta\left(\frac{1+\nu}{2}, \frac{1}{2}\right)\right)^{n_c}}{\beta\left(\frac{n_c+\nu}{2}, \frac{n_c}{2}\right)} \right] + \frac{n_c(1+\nu)}{2} \left[\psi\left(\frac{1+\nu}{2}\right) - \psi\left(\frac{\nu}{2}\right) \right] \\ & - \frac{n_c + \nu}{2} \left[\psi\left(\frac{n_c + \nu}{2}\right) - \psi\left(\frac{\nu}{2}\right) \right], \end{aligned} \tag{S1-36}$$

with β being the beta-function, and ψ the digamma-function. Note that the matrix $\mathbf{\Lambda}$ is the inverse of the matrix A used by [54]. Since mutual information is scale invariant, $\mathbf{s}_k$ has the mutual information in (S1-35). For the case of $n_c = 2$, $\log |\mathbf{\Lambda}_k| = -\log(1 - \rho_k^2)$ which, together with (S1-35), yields (13) in the main text.

S1.3 Relation to canonical correlation analysis

We consider here the case of two data sets and derive (6). We start with computing $1/(\nu_k - 2)\mathbf{y}_k^\top \mathbf{\Lambda}_k \mathbf{y}_k$. Using the definition of $\mathbf{y}_k$,

$$\mathbf{y}_k = (\langle \mathbf{q}_k^{\mathrm{A}}, \mathbf{z}^{\mathrm{A}} \rangle,\ \langle \mathbf{q}_k^{\mathrm{D}}, \mathbf{z}^{\mathrm{D}} \rangle)^\top, \tag{S1-37}$$

and the definition of $\mathbf{\Lambda}_k$ in (3), we obtain

$$\frac{\mathbf{y}_k^\top \mathbf{\Lambda}_k \mathbf{y}_k}{\nu_k - 2} = \frac{1}{\nu_k - 2} \frac{1}{1 - \rho_k^2} \left[\langle \mathbf{q}_k^{\mathrm{A}}, \mathbf{z}^{\mathrm{A}} \rangle^2 + \langle \mathbf{q}_k^{\mathrm{D}}, \mathbf{z}^{\mathrm{D}} \rangle^2 - 2\rho_k \langle \mathbf{q}_k^{\mathrm{A}}, \mathbf{z}^{\mathrm{A}} \rangle \langle \mathbf{q}_k^{\mathrm{D}}, \mathbf{z}^{\mathrm{D}} \rangle \right]. \tag{S1-38}$$

For large ν_k the term $1/(\nu_k - 2)\mathbf{y}_k^\top \mathbf{\Lambda}_k \mathbf{y}_k$ is small. Hence,

$$\log\left(1 + \frac{1}{\nu_k - 2} \mathbf{y}_k^\top \mathbf{\Lambda}_k \mathbf{y}_k\right) = \frac{1}{\nu_k - 2} \mathbf{y}_k^\top \mathbf{\Lambda}_k \mathbf{y}_k + O\left(\frac{1}{\nu_k^2}\right), \tag{S1-39}$$

where we have used the first-order Taylor expansion of $\log(1+x)$ around $x=0$. Dropping terms of order $1/\nu_k^2$ and smaller, we have for $f(\mathbf{Q}^{\mathrm{A}}, \mathbf{Q}^{\mathrm{D}})$ in (5)

$$f(\mathbf{q}_1^{\mathrm{A}}, \dots, \mathbf{q}_m^{\mathrm{D}}) \approx \mathrm{const} - \frac{1}{T}\sum_{t=1}^{T}\sum_{k=1}^{m} \frac{\nu_k+2}{2\nu_k-4}\frac{1}{1-\rho_k^2}\left[\langle \mathbf{q}_k^{\mathrm{A}}, \mathbf{z}^{\mathrm{A}}(t)\rangle^2 + \langle \mathbf{q}_k^{\mathrm{D}}, \mathbf{z}^{\mathrm{D}}(t)\rangle^2 - 2\rho_k \langle \mathbf{q}_k^{\mathrm{A}}, \mathbf{z}^{\mathrm{A}}(t)\rangle\langle \mathbf{q}_k^{\mathrm{D}}, \mathbf{z}^{\mathrm{D}}(t)\rangle\right]. \tag{S1-40}$$

Since ν_k is assumed large,

$$\frac{\nu_k+2}{2\nu_k-4} \approx \frac{1}{2} \tag{S1-41}$$

and thus

$$f(\mathbf{q}_1^{\mathrm{A}}, \dots, \mathbf{q}_m^{\mathrm{D}}) \approx \mathrm{const} - \frac{1}{T}\sum_{t=1}^{T}\sum_{k=1}^{m} \frac{1}{2}\frac{1}{1-\rho_k^2}\left[\langle \mathbf{q}_k^{\mathrm{A}}, \mathbf{z}^{\mathrm{A}}(t)\rangle^2 + \langle \mathbf{q}_k^{\mathrm{D}}, \mathbf{z}^{\mathrm{D}}(t)\rangle^2 - 2\rho_k \langle \mathbf{q}_k^{\mathrm{A}}, \mathbf{z}^{\mathrm{A}}(t)\rangle\langle \mathbf{q}_k^{\mathrm{D}}, \mathbf{z}^{\mathrm{D}}(t)\rangle\right]. \tag{S1-42}$$

The sum over the samples is

$$\sum_{t=1}^{T}\langle \mathbf{q}_k^{\mathrm{A}}, \mathbf{z}^{\mathrm{A}}(t)\rangle^2 + \langle \mathbf{q}_k^{\mathrm{D}}, \mathbf{z}^{\mathrm{D}}(t)\rangle^2 - 2\rho_k \langle \mathbf{q}_k^{\mathrm{A}}, \mathbf{z}^{\mathrm{A}}(t)\rangle\langle \mathbf{q}_k^{\mathrm{D}}, \mathbf{z}^{\mathrm{D}}(t)\rangle,$$

which equals

$$T\left[\mathbf{q}_k^{\mathrm{A}\top}\hat{\mathbf{K}}_{\mathrm{A}}\mathbf{q}_k^{\mathrm{A}} + \mathbf{q}_k^{\mathrm{D}\top}\hat{\mathbf{K}}_{\mathrm{D}}\mathbf{q}_k^{\mathrm{D}} - 2\rho_k \mathbf{q}_k^{\mathrm{D}\top}\hat{\mathbf{K}}_{\mathrm{DA}}\mathbf{q}_k^{\mathrm{A}}\right],$$

where the matrices

$$\hat{\mathbf{K}}_{\mathrm{A}} = \frac{1}{T}\sum_{t=1}^{T}\mathbf{z}^{\mathrm{A}}(t)\mathbf{z}^{\mathrm{A}}(t)^\top, \qquad \hat{\mathbf{K}}_{\mathrm{D}} = \frac{1}{T}\sum_{t=1}^{T}\mathbf{z}^{\mathrm{D}}(t)\mathbf{z}^{\mathrm{D}}(t)^\top, \qquad \hat{\mathbf{K}}_{\mathrm{DA}} = \frac{1}{T}\sum_{t=1}^{T}\mathbf{z}^{\mathrm{D}}(t)\mathbf{z}^{\mathrm{A}}(t)^\top \tag{S1-43}$$

are the sample covariance matrices and the cross-correlation matrix of $\mathbf{z}^{\mathrm{D}}$ and $\mathbf{z}^{\mathrm{A}}$. The matrices $\hat{\mathbf{K}}_{\mathrm{A}}$ and $\hat{\mathbf{K}}_{\mathrm{D}}$ are the identity by the assumed preprocessing. Since $\mathbf{q}_k^{\mathrm{A}}$ and $\mathbf{q}_k^{\mathrm{D}}$ are the columns of an orthonormal matrix, we obtain for all k

$$\mathbf{q}_k^{\mathrm{A}\top}\hat{\mathbf{K}}_{\mathrm{A}}\mathbf{q}_k^{\mathrm{A}} = 1, \qquad \mathbf{q}_k^{\mathrm{D}\top}\hat{\mathbf{K}}_{\mathrm{D}}\mathbf{q}_k^{\mathrm{D}} = 1. \tag{S1-44}$$

We obtain (6) by plugging these relations into (S1-42),

$$f(\mathbf{q}_1^{\mathrm{A}}, \dots, \mathbf{q}_m^{\mathrm{D}}) \approx \mathrm{const} - \sum_{k=1}^{m}\frac{1}{1-\rho_k^2}\frac{1}{2}\left[2 - 2\rho_k \mathbf{q}_k^{\mathrm{D}\top}\hat{\mathbf{K}}_{\mathrm{DA}}\mathbf{q}_k^{\mathrm{A}}\right] \tag{S1-45}$$

$$\approx \mathrm{const} + \sum_{k=1}^{m}\frac{1}{1-\rho_k^2}\left[\rho_k \mathbf{q}_k^{\mathrm{D}\top}\hat{\mathbf{K}}_{\mathrm{DA}}\mathbf{q}_k^{\mathrm{A}}\right]. \tag{S1-46}$$
