# Supplementary material for: Spatio-Chromatic Adaptation via Higher-Order Canonical Correlation Analysis of Natural Images
Source: PLoS One. 2014 Feb 12;9(2):e86481. doi: 10.1371/journal.pone.0086481 (PMC3922757; doi:10.1371/journal.pone.0086481)
Supplement: Text S2 — Background Material from Multivariate Analysis. (PDF) [file pone.0086481.s002.pdf]

S2 Background Material from Multivariate Analysis

We present here background material from multivariate analysis that is relevant for this paper. In Section S2.1, we briefly review whitening and dimension reduction by principal component analysis (PCA). Section S2.2 is on regression and how to use it in order to determine the amount of dimension reduction. In Section S2.3, we review canonical correlation analysis. For more background than provided here, we refer the reader to Chapters 3 and 14 of [22].

S2.1 Whitening and PCA dimension reduction

Both whitening of zero mean $\mathbf{x}^{\mathrm{A}} \in \mathrm{R}^{n^{\mathrm{A}}}, \mathbf{x}^{\mathrm{D}} \in \mathrm{R}^{n^{\mathrm{D}}}$ and reducing their dimension by PCA to $m^{\mathrm{A}}, m^{\mathrm{D}}$ can be performed by

$$\mathbf{z}^{\mathrm{A}} = \mathbf{V}^{\mathrm{A}}\mathbf{x}^{\mathrm{A}}, \qquad \mathbf{z}^{\mathrm{D}} = \mathbf{V}^{\mathrm{D}}\mathbf{x}^{\mathrm{D}}, \tag{S2-1}$$

where $\mathbf{V}^{\mathrm{A}}$ and $\mathbf{V}^{\mathrm{D}}$ are $m^{\mathrm{A}} \times n^{\mathrm{A}}$ and $m^{\mathrm{D}} \times n^{\mathrm{D}}$ whitening matrices,

$$\mathbf{V}^{\mathrm{A}} = (\mathbf{D}^{\mathrm{A}})^{-1/2}(\mathbf{E}^{\mathrm{A}})^{\top}, \qquad \mathbf{V}^{\mathrm{D}} = (\mathbf{D}^{\mathrm{D}})^{-1/2}(\mathbf{E}^{\mathrm{D}})^{\top}. \tag{S2-2}$$

The diagonal matrices $\mathbf{D}^{\mathrm{A}}$ and $\mathbf{D}^{\mathrm{D}}$ contain the m^{A} and m^{D} largest eigenvalues of the covariance matrix of $\mathbf{x}^{\mathrm{A}}$ and $\mathbf{x}^{\mathrm{D}}$, respectively. The matrices $\mathbf{E}^{\mathrm{A}}$ and $\mathbf{E}^{\mathrm{D}}$ have as columns the corresponding eigenvectors. The (pseudo) inverses of $\mathbf{V}^{\mathrm{A}}$ and $\mathbf{V}^{\mathrm{D}}$ are the matrices $(\mathbf{V}^{\mathrm{A}})^{\dagger} = \mathbf{E}^{\mathrm{A}}(\mathbf{D}^{\mathrm{A}})^{1/2}$ and $(\mathbf{V}^{\mathrm{D}})^{\dagger} = \mathbf{E}^{\mathrm{D}}(\mathbf{D}^{\mathrm{D}})^{1/2}$, respectively.

S2.2 Regression to determine the degree of dimension reduction

For zero mean random variables, the linear prediction of $\mathbf{x}^{\mathrm{D}}$ from $\mathbf{x}^{\mathrm{A}}$ which minimizes the expected squared error is given by $\hat{\mathbf{x}}^{\mathrm{D}} = \mathbf{B}\mathbf{x}^{\mathrm{A}}$, with regression matrix $\mathbf{B}$,

$$\mathbf{B} = \mathrm{E}(\mathbf{x}^{\mathrm{D}}\mathbf{x}^{\mathrm{A}\top})\left[\mathrm{E}(\mathbf{x}^{\mathrm{A}}\mathbf{x}^{\mathrm{A}\top})\right]^{-1}, \tag{S2-3}$$

where we assume that the covariance matrix $\mathrm{E}(\mathbf{x}^{\mathrm{A}}\mathbf{x}^{\mathrm{A}\top})$ is invertible. If $\mathrm{E}(\mathbf{x}^{\mathrm{A}}\mathbf{x}^{\mathrm{A}\top})$ is invertible but badly conditioned, taking the inverse is a nonrobust operation. The matrix is badly conditioned if the components of $\mathbf{x}^{\mathrm{A}}$ are strongly correlated, so that only few data points lie outside a subspace of lower dimensionality than n^{A}. This means that only few data points determine the behavior of $\mathbf{B}$ outside that subspace. As a consequence, the variance of the prediction (the prediction error) can get large. Reducing the dimension of the data prior to the regression may reduce the prediction error. However, if too many dimensions are omitted the prediction error increases. There is thus an optimal amount of dimension reduction. It can be found empirically by comparing the prediction error for different numbers of retained dimensions.

In the main part of the paper, $\mathbf{x}^{\mathrm{A}}$ and $\mathbf{x}^{\mathrm{D}}$ are of the same dimensionality n. Since they show the same physical objects, we reduced the dimension of both data sets by the same amount using (S2-1), with $m^{\mathrm{A}} = m^{\mathrm{D}} = m$. The regression matrix for the whitened and dimension reduced data is the $m \times m$ matrix $\mathbf{K}_{\mathrm{DA}}$,

$$\mathbf{K}_{\mathrm{DA}} = \mathrm{E}(\mathbf{z}^{\mathrm{D}}\mathbf{z}^{\mathrm{A}\top})\left[\mathrm{E}(\mathbf{z}^{\mathrm{A}}\mathbf{z}^{\mathrm{A}\top})\right]^{-1} = \mathrm{E}(\mathbf{z}^{\mathrm{D}}\mathbf{z}^{\mathrm{A}\top}), \tag{S2-4}$$

which is the cross-correlation matrix between $\mathbf{z}^{\mathrm{D}}$ and $\mathbf{z}^{\mathrm{A}}$. Including the whitening matrices into the formula yields the rank m regression matrix $\mathbf{B}_m$ for the prediction of $\mathbf{x}^{\mathrm{D}}$ from $\mathbf{x}^{\mathrm{A}}$,

$$\mathbf{B}_m = (\mathbf{V}^{\mathrm{D}})^{\dagger}\mathbf{K}_{\mathrm{DA}}\mathbf{V}^{\mathrm{A}}. \tag{S2-5}$$

We measured the prediction error using the coefficient of determination R^2 on test data,

$$R^2(m) = 1 - \frac{\text{average squared prediction error of } \mathbf{x}^{\mathrm{D}} \text{ using } \mathbf{B}_m}{\text{total variance of } \mathbf{x}^{\mathrm{D}}}, \tag{S2-6}$$

and set the number of dimensions retained in (S2-1) to the value of m which minimized R^2.

S2.3 Canonical correlation analysis

Canonical correlation analysis (CCA) is a classical method to find related features in two data sets, that is, the matrices $\mathbf{Q}^{\mathrm{A}}$ and $\mathbf{Q}^{\mathrm{D}}$ in Figure 3. In CCA, related means correlated. After whitening and, possibly, dimension reduction, CCA rotates the individual coordinate systems of data $\mathbf{z}^{\mathrm{A}}$ and $\mathbf{z}^{\mathrm{D}}$ such that the corresponding coordinates s_k^{A} and s_k^{D} are maximally correlated. If $\mathbf{z}^{\mathrm{A}}$ and $\mathbf{z}^{\mathrm{D}}$ have dimensions m^{A} and m^{D}, respectively, CCA allows to find $m = \min(m^{\mathrm{A}}, m^{\mathrm{D}})$ related features. The features are found by the singular value decomposition of the cross-correlation matrix $\mathbf{K}_{\mathrm{DA}}$ between $\mathbf{z}^{\mathrm{D}}$ and $\mathbf{z}^{\mathrm{A}}$,

$$\mathbf{K}_{\mathrm{DA}} = \mathbf{Q}^{\mathrm{D}}\mathbf{S}(\mathbf{Q}^{\mathrm{A}})^{\top}. \tag{S2-7}$$

The matrix $\mathbf{S}$ is diagonal and contains the correlation coefficients between the canonical coordinates s_k^{A} and s_k^{D}. The $m^{\mathrm{D}} \times m$ and $m^{\mathrm{A}} \times m$ matrices $\mathbf{Q}^{\mathrm{D}}$ and $\mathbf{Q}^{\mathrm{A}}$ contain the features which have maximally correlated canonical coordinates. CCA is insensitive to statistical dependencies beyond correlation, both across and within the data sets. From Section S2.2, it follows that CCA is closely connected to linear regression.
