# Supplementary material for: Spatio-Chromatic Adaptation via Higher-Order Canonical Correlation Analysis of Natural Images
Source: PLoS One. 2014 Feb 12;9(2):e86481. doi: 10.1371/journal.pone.0086481 (PMC3922757; doi:10.1371/journal.pone.0086481)
Supplement: Text S3 — Calculations for the Noise-Distortion Analysis. (PDF) [file pone.0086481.s003.pdf]

S3 Calculations for the Noise-Distortion Analysis

This section contains the detailed calculations to derive the analytical expression in (7) for the squared prediction error $\mathrm{E}\,||\mathbf{s}^{\mathrm{D}} - \tilde{\mathbf{s}}^{\mathrm{D}}||^2$.

The squared error of the prediction is

$$\begin{aligned} \mathrm{E}\left(||\mathbf{s}^{\mathrm{D}} - \tilde{\mathbf{s}}^{\mathrm{D}}||^2\right) &= \mathrm{E}\left(\sum_k (s_k^{\mathrm{D}} - \hat{s}_k^{\mathrm{D}} - \sigma(\hat{s}_k^{\mathrm{D}})n_k)^2\right) && \text{(S3-1)} \\ &= \sum_k \mathrm{E}\left[(s_k^{\mathrm{D}} - \hat{s}_k^{\mathrm{D}})^2 + \sigma^2(\hat{s}_k^{\mathrm{D}})n_k^2 - 2(s_k^{\mathrm{D}} - \hat{s}_k^{\mathrm{D}})\sigma(\hat{s}_k^{\mathrm{D}})n_k\right] && \text{(S3-2)} \\ &= \sum_k \mathrm{E}\left[(s_k^{\mathrm{D}} - \hat{s}_k^{\mathrm{D}})^2 + \sigma^2(\hat{s}_k^{\mathrm{D}})n_k^2\right], && \text{(S3-3)} \end{aligned}$$

where the last equation follows from the zero mean and independence assumption for n_k. Using the definition of $\sigma^2(\hat{s}_k^{\mathrm{D}})$, and the independence assumption for n_k, we have

$$\mathrm{E}\left(||\mathbf{s}^{\mathrm{D}} - \tilde{\mathbf{s}}^{\mathrm{D}}||^2\right) = \sum_k \mathrm{E}\left[(s_k^{\mathrm{D}} - \hat{s}_k^{\mathrm{D}})^2\right] + F\,\mathrm{E}\left(|\hat{s}_k^{\mathrm{D}}|\right)\mathrm{E}(n_k^2). \quad \text{(S3-4)}$$

The variance of n_k is one so that

$$\mathrm{E}\left(||\mathbf{s}^{\mathrm{D}} - \tilde{\mathbf{s}}^{\mathrm{D}}||^2\right) = \mathrm{E}\left(||\mathbf{s}^{\mathrm{D}} - \hat{\mathbf{s}}^{\mathrm{D}}||\right) + F\sum_k \mathrm{E}\left(|\hat{s}_k^{\mathrm{D}}|\right). \quad \text{(S3-5)}$$

Using that $\hat{s}_k^{\mathrm{D}} = \varrho_k s_k^{\mathrm{A}}$, we obtain (7),

$$\mathrm{E}\left(||\mathbf{s}^{\mathrm{D}} - \tilde{\mathbf{s}}^{\mathrm{D}}||^2\right) = \mathrm{E}\left(||\mathbf{s}^{\mathrm{D}} - \hat{\mathbf{s}}^{\mathrm{D}}||^2\right) + F\sum_k |\varrho_k|\,\mathrm{E}\left(|s_k^{\mathrm{A}}|\right). \quad \text{(S3-6)}$$
